# Particle Simulation of Oxidation Induced Band 3 Clustering in Human Erythrocytes

**DOI:** 10.1371/journal.pcbi.1004210

**Published:** 2015-06-05

**Authors:** Hanae Shimo, Satya Nanda Vel Arjunan, Hiroaki Machiyama, Taiko Nishino, Makoto Suematsu, Hideaki Fujita, Masaru Tomita, Koichi Takahashi

**Affiliations:** 1 Laboratory for Biochemical Simulation, RIKEN Quantitative Biology Center, Osaka, Japan; 2 Department of Biochemistry, School of Medicine, Keio University, Shinjuku, Tokyo, Japan; 3 Immunology Frontier Research Center, Osaka University, Osaka, Japan; 4 Institute for Advanced Biosciences, Keio University, Tsuruoka, Yamagata, Japan; 5 Department of Environment and Information Studies, Keio University, Fujisawa, Kanagawa, Japan; University of Michigan, UNITED STATES

## Abstract

Oxidative stress mediated clustering of membrane protein band 3 plays an essential role in the clearance of damaged and aged red blood cells (RBCs) from the circulation. While a number of previous experimental studies have observed changes in band 3 distribution after oxidative treatment, the details of how these clusters are formed and how their properties change under different conditions have remained poorly understood. To address these issues, a framework that enables the simultaneous monitoring of the temporal and spatial changes following oxidation is needed. In this study, we established a novel simulation strategy that incorporates deterministic and stochastic reactions with particle reaction-diffusion processes, to model band 3 cluster formation at single molecule resolution. By integrating a kinetic model of RBC antioxidant metabolism with a model of band 3 diffusion, we developed a model that reproduces the time-dependent changes of glutathione and clustered band 3 levels, as well as band 3 distribution during oxidative treatment, observed in prior studies. We predicted that cluster formation is largely dependent on fast reverse reaction rates, strong affinity between clustering molecules, and irreversible hemichrome binding. We further predicted that under repeated oxidative perturbations, clusters tended to progressively grow and shift towards an irreversible state. Application of our model to simulate oxidation in RBCs with cytoskeletal deficiency also suggested that oxidation leads to more enhanced clustering compared to healthy RBCs. Taken together, our model enables the prediction of band 3 spatio-temporal profiles under various situations, thus providing valuable insights to potentially aid understanding mechanisms for removing senescent and premature RBCs.

## Author Summary

In order to maintain a steady internal environment, our bodies must be able to specifically recognize old and damaged red blood cells (RBCs), and remove them from the circulation in a timely manner. Clusters of membrane protein band 3, which form in response to elevated oxidative damage, serve as essential molecular markers that initiate this cell removal process. However, little is known about the details of how these clusters are formed and how their properties change under different conditions. To understand these mechanisms in detail, we developed a computational model that enables the prediction of the time course profiles of metabolic intermediates, as well as the visualization of the resulting band 3 distribution during oxidative treatment. Our model predictions were in good agreement with previous published experimental data, and provided predictive insights on the key factors of cluster formation. Furthermore, simulation experiments of the effects of multiple oxidative pulses and cytoskeletal defect using the model also suggested that clustering is enhanced under such conditions. Analyses using our model can provide hypotheses and suggest experiments to aid the understanding of the physiology of anemia-associated RBC disorders, and optimization of quality control of RBCs in stored blood.

## Introduction

The clustering of membrane proteins plays an important role in various cellular processes, ranging from signal transduction to cell migration [1]. In human red blood cells (RBCs), oxidation induced clustering of anion exchanger 1 (band 3) greatly contributes to determining the timing of cell removal, by generating a high affinity site for autologous antibody binding. Although enhanced band 3 clustering has been closely associated with certain RBC disorders causing hemolytic anemia, such as glucose-6-phosphate dehydrogenase (G6PD) deficiency [2,3], malaria [4,5], and sickle-cell disease [6], as well as critical biochemical changes during blood storage [7,8], the details of its molecular mechanisms remain poorly understood.

Band 3 is an integral membrane protein that accounts for approximately 25% of the RBC membrane surface. It has a number of functions, including the aid of anion transport across the membrane [9,10], regulation of the glycolytic pathway [11,12], stabilization of the membrane structure [13,14], and control of RBC lifespan [15,16]. At normal state, several band 3 are bound to a cytoskeletal network of spectrin, a long, flexible rod protein, and have limited or confined diffusion. However, elevation of oxidative stress levels, promotes the oxidation [17], phosphorylation [18], and dissociation of band 3 from the spectrin cytoskeleton [19], resulting in enhanced mobility. This subsequently leads to the formation of band 3 clusters, which form a neoantigen that binds autologous immunoglobulin (IgG) and complement, thus allowing opsonization or direct recognition by phagocytes [20,21].

Several experimental studies in the past have attempted to characterize the chain of reactions leading to band 3 clustering, using *in vitro* models [3,4]. Increased oxidation, phosphorylation, and clustered band 3 levels have been observed in such experiments of RBCs treatment with oxidizing agent diamide. While inhomogeneous distributions of band 3 have also been observed in other studies [3,7,22–25], there has been no framework to integrate such spatial and temporal data, to enable the simultaneous monitoring of multiple factors during oxidative treatment. Moreover, utilizing single-particle tracking techniques using fluorescent protein tags for direct measurement of its reaction dynamics, such as in other protein clustering studies [26,27], remains difficult because of the lack of protein synthesis machinery and large quantity of band 3 in RBCs. Thus, details of how these clusters form, and how they behave under different conditions remains largely unexplored.

Computational models have the advantage of enabling observation of the time profile of multiple components, and the effects of changes in multiple individual parameters on the total system. Due to the pivotal role of RBCs in our bodies and the simplicity of their systems, a number of models of human RBC biochemical pathways have been published in the past [28–32]. Previously, we developed a computational model to assess the changes in RBC metabolism during oxidation by hydrogen peroxide [33], however spatial information and reactions representing alterations in band 3 were excluded. To mechanistically describe oxidation induced band 3 clustering, a novel strategy that incorporates deterministic algorithms to model metabolic reactions, and stochastic algorithms with particle reaction-diffusion processes to model band 3 behavior is needed.

**Fig 1:**
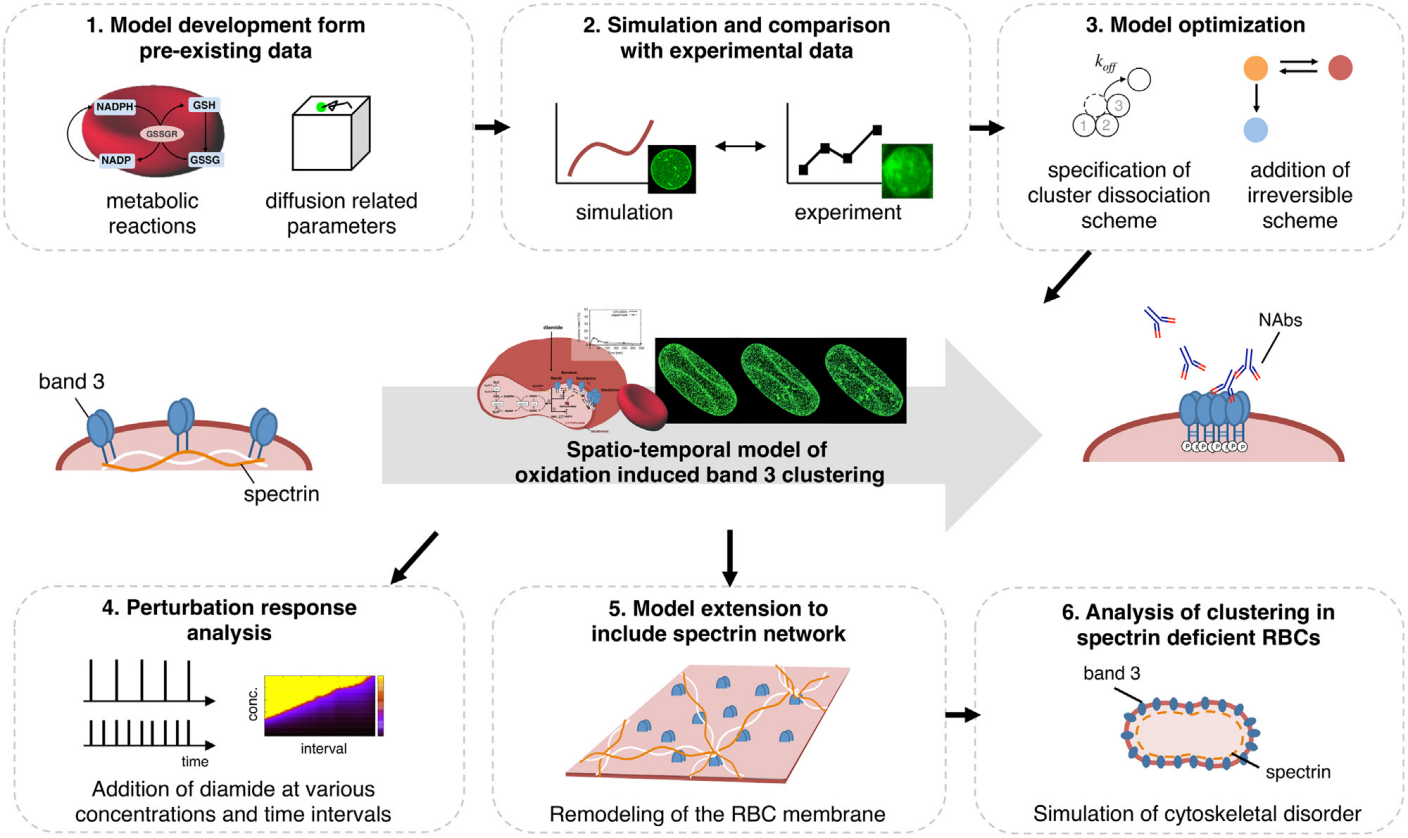
Workflow diagram of present study. We developed a particle model to represent the spatial and temporal changes that occur during RBC oxidation, which result in the clustering of band 3 and opsonization by naturally occurring antibodies (NAbs). (1) Major reactions from a prior kinetic model of RBC metabolism [39], and experimentally validated diffusion related parameters for band 3 were integrated into a single-particle simulation model. (2) Time-course data and visual data resulting from simulations under 0.25 mM diamide treatment were compared with those of past experiments. (3) The model was optimized by testing various reaction schemes, including specification of cluster dissociation rates and the addition of an irreversible clustering reaction. (4) The model was simulated under multiple diamide additions for varying concentrations and time intervals. (5) An extended model including physical confinement by spectrin cytoskeletal components was developed. (6) The spectrin model was applied to assess band 3 clustering in spectrin deficient RBCs.

By integrating a kinetic model of RBC antioxidant metabolism with a model of band 3 diffusion, we introduce a particle simulation model that enables the prediction of oxidation induced band 3 cluster formation at single molecule resolution (overview illustrated in Fig 1). We show that our model reproduces the time-dependent changes of glutathione (GSH) and clustered band 3 levels, as well as band 3 distribution in human RBCs treated with diamide, observed in experimental studies. Through parameter analysis, we predict that the formation of these transient and densely clustered regions of band 3, are dependent on high affinity between clustered molecules and irreversible state transitions of band 3. In addition, we simulate the responses of band 3 under repeated oxidative perturbation, to predict how clustering could contribute to *in vivo* erythrophagocytosis. Finally, we extend the model to include to the effects of cytoskeletal components, to observe how functional impairment of the membrane cytoskeleton could affect band 3 clustering.

## Materials and Methods

### Simulation software and technology

Our model was developed on E-Cell Simulation Environment Version 3 (E-Cell 3) [34–36] installed with Spatiocyte [37]. Spatiocyte is a lattice-based Monte-Carlo simulation method that can model complex reaction-diffusion mediated cellular processes at single molecule resolution. To represent cell compartments and to rapidly resolve molecular collisions, the method discretizes the three-dimensional space into hexagonal closed-packed lattice. Each molecule randomly walks voxel-to-voxel in a time step, calculated from its diffusion coefficient. Molecular collisions can take place between each walk. Collisions between two reactive species molecules can generate one or two product molecules with a probability, *p* that is computed from the rate of reaction. Immobile lipid molecules represent surface compartments such as cellular and nuclear membranes. Stochastic reactions involving homogeneously distributed species in a compartment are performed using a modified Next Reaction method [37,38]. In this work, we have further integrated the Spatiocyte method with the ordinary differential equation (ODE) solvers of E-Cell 3 to simultaneously perform Michaelis-Menten and mass action reactions involving large number of metabolites. The integration of stochastic, deterministic and particle reactions with the diffusion processes of Spatiocyte was supported by the multi-algorithm, multi-timescale method of E-Cell 3.

### Overall model structure

**Fig 2:**
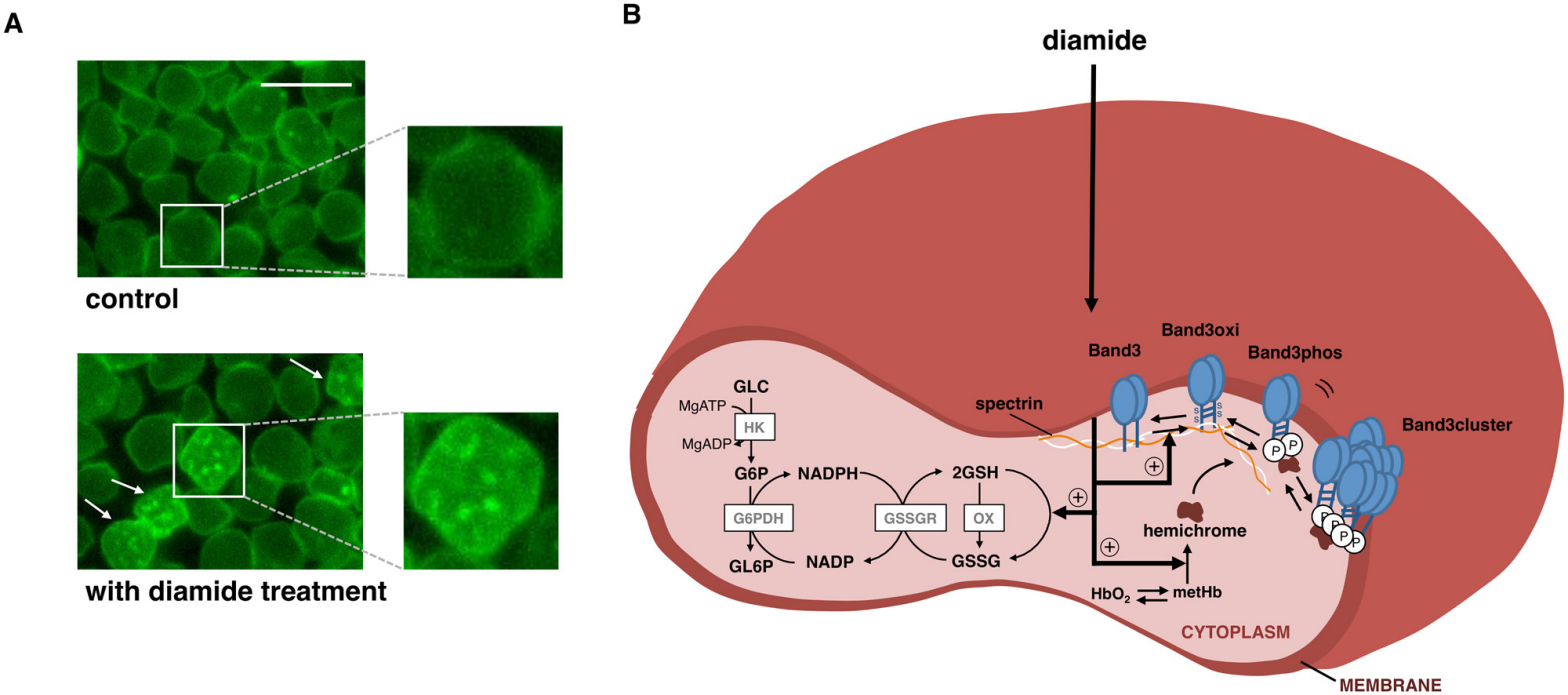
Remodeling of diamide induced band 3 cluster formation in RBCs. (A) A representative image of band 3 distribution in control and diamide treated mouse RBCs obtained by epifluorescence microscopy. Scale bar is 10 μm, inset represents a higher magnification of the portion of the cell indicated. Arrows indicate dense cluster-like dense regions of band 3 on the membrane. (B) Schematic representation of the band 3 clustering model. Nodes and edges in the cytoplasm represent metabolites/hemoglobin states and enzymatic reactions, respectively. Nodes in the membrane indicate diffusion-reaction processes. Bold arrows represent diamide-mediated reactions. Abbreviations: Glucose; GLC, Glucose-6-phosphate; G6P, Glucose-6-phosphatase; GL6P, Reduced nicotinamide adenine dinucleotide phosphate; NADPH, Nicotinamide adenine dinucleotide phosphate; NADP, Reduced glutathione; GSH, Oxidized glutathione; GSSG, Adenosine triphosphate; MgATP, Adenosine diphosphate; MgADP, Phosphate group; P, Oxygenated hemoglobin; HbO_2_, Methemoglobin; metHb, Denatured hemoglobin; hemichrome, Hexokinase; HK, Glucose-6-phosphate dehydrogenase; G6PDH, glutathione reductase; GSSGR, Glutathione turnover; OX, Oxidized band 3; Band3oxi, Phosphorylated band 3; Band3phos, Clustered band 3; Band3cluster.

A schematic representation of our developed model and example images of RBCs exhibiting inhomogeneous distribution of band 3 in presence of diamide are shown in Fig 2. Reaction rates and parameters of the antioxidant pathways were extracted from a previous whole human RBC metabolism model [39]. The full set of the ODEs, parameter list, and initial conditions for these reactions are provided in S1 Text. Since the focus of this study is to reproduce the effects of oxidation at single molecule resolution, rather than precisely predict the metabolic changes, a minimal amount of metabolic pathways was implemented into the model.

Reaction-diffusion of band 3 was modeled using Spatiocyte. The series of band 3 related reactions is given in Table 1. The initial model parameters and reactions rates for these reactions are given in S1 Table. *D*_*i*_ represents the diffusion coefficient of molecule *i*, *k* represents intrinsic rate constants, and *p* represents the probability for a reactive collision between reactants. Reaction rates and schemes for band 3 oxidation, phosphorylation and clustering were determined by manually fitting the GSH depletion and reversible band 3 clustering curve in control RBCs, as measured by a previous experiment [3]. Cluster images, and cluster size estimates [23] from previous experimental studies, were also used to estimate the parameters that were not available from literature. All simulation results represent the average for stochastic simulations of 100 runs.

**Table 1:** Model reactions.

#	Equation	Rate	Probability	Reference
1	Diamide + Band3 → Band3oxi	1.0 × 10^–23^ m s^-1^	—	fitted to [3]
2	Diamide + 2GSH → GSSG	300 M^-1^ s^-1^	—	[41]
3	Band3oxi + 2GSH → Band3 + GSSG	1.0 × 10^–50^ m^3^ s^-1^	—	fitted to [3]
4	Band3oxi → Band3phos	5.0 × 10^–4^ s^-1^	—	fitted to [3]
5	Band3phos → Band3oxi	5.0 × 10^–3^ s^-1^	—	fitted to [3]
6	Band3phos + Band3phos → Band3cluster + Band3cluster	3.45 × 10^–13^ m^3^ s^-1^	0.1	fitted to [3]
7	Band3phos + Band3cluster → Band3cluster + Band3cluster	3.45 × 10^–12^ m^3^ s^-1^	1	fitted to [3]
8	Band3cluster → Band3phos	4^(6-n)^ s^-1^ for n bound sites	—	fitted to [3]
9	HbO_2_ + diamide → hemichrome	KmS: 1.0 × 10^–4^, KcF: 1.0 × 10^–3^	—	fitted to [70]
10	Band3oxi + hemichrome → hemiBand3oxi	1.0 × 10^–23^ m s^-1^	—	fitted to [3]
11	hemiBand3oxi →hemiBand3oxi	5.0 × 10^–4^ s^-1^	—	fitted to [3]
12	hemiBand3phos + hemiBand3phos → hemiBand3cluster + hemiBand3cluster	1.03 × 10^–12^ m^3^ s^-1^	0.3	fitted to [3]
	Band3phos + hemiBand3phos → Band3cluster + hemiBand3cluster	1.03 × 10^–12^ m^3^ s^-1^	0.3	fitted to [3]
13	hemiBand3phos + hemiBand3cluster → hemiBand3cluster + hemi Band3cluster	3.45 × 10^–12^ m^3^ s^-1^	1	fitted to [3]
	Band3phos + hemiBand3cluster → Band3cluster + hemi Band3cluster	3.45 × 10^–12^ m^3^ s^-1^	1	fitted to [3]
	hemiBand3phos + Band3cluster → hemiBand3cluster + Band3cluster	3.45 × 10^–12^ m^3^ s^-1^	1	fitted to [3]
14	hemiBand3cluster → hemiBand3phos	2^(6-n)^ s^-1^ for n bound sites	—	fitted to [3]
15	hemiBand3phos → hemiBandoxi	5.0 × 10^–3^ s^-1^	—	fitted to [3]

### Description of the band 3 clustering reactions

Our model is composed of largely three components of the band 3 clustering process; oxidation, phosphorylation, and clustering. The process of oxidation is represented by the formation of oxidized band 3 (Band3oxi; Table 1 equation 1), which is based on previous evidence of oxidized—SH groups in band 3 in the presence of diamide [40]. Further, we have also included diamide induced rapid conversion of GSH to oxidized glutathione (GSSG) ([41], Table 1 equation 2) and the formation of mixed disulfides between GSH and other protein—SH groups to form S-glutathionylated proteins (PSSG, [42,43]; S1 Text) in the model. Band 3 oxidation is modeled as a reversible reaction, as it has been previously reported that GSH also promotes the reduction of oxidized band 3, returning it to the normal state (Table 1 equation 3).

The binding and phosphorylation of the band 3 cytoplamic region by Syk tyrosine kinase, has been previously shown to follow oxidation (Table 1 equation 4). In the model we have simplified the formation of phosphorylated band 3 (Band3phos) to be independent of Syk concentration, based on the assumption that Syk exists in sufficient amounts throughout the interior of the RBC. As band 3 has previously been identified as a target for SHP-2 tyrosine phosphatase [44], we also included its dephosphorylation reaction (Table 1 equation 5).

In the actual RBC, it has been reported that band 3 bound to the spectrin cytoskeleton, detaches from spectrin upon phosphorylation, and gains greater mobility. In our main model we have represented this with an increase in diffusion coefficient, as observed by an experimental study. [19]. We mimicked cluster formation by creating an immobile cluster species (Band3cluster) that forms as phosphorylated band 3 molecules collide. The immobility of Band3cluster is based on previous observations of the static clustered proteins [26]. The clustering process was separated into two parts; the initial nucleation of the cluster (Table 1 equation 6), and the following growth of the cluster (Table 1 equation 7). As reversible clustering has been described in previous works, we also included a reaction that allows clustered molecules to dissociate from the cluster at certain rates (Table 1, equation 8). Furthermore, in addition to these basal reactions, we added a scheme for the attachment of hemichrome, a degraded product of hemoglobin (Hb) oxidation (Table 1 equation 9–15). This will be further discussed in the following sections.

### Simulation of oxidation by diamide treatment

All simulations were carried out on a Linux cluster, running E-Cell 3 and Spatiocyte. A single molecule was set to represent a dimeric band 3 in its natural form [45]. During initialization, all band 3 molecules were randomly placed on the cuboid compartment surface with a dimension of 1.06 μm length, 1.06 μm width, and 89 nm height (S1 Table), which represents approximately one-thousandth volume of an actual RBC and the corresponding surface area. Simulations were performed using voxels of radius 3.62 nm, and for this setup with 4800 diffusing molecules, it took approximately five hours of actual time to simulate six hours. To compare the localization of multiple clusters on a whole RBC surface, additional simulations were also run on a RBC-like biconcave compartment (S1 Text) with one-hundredth volume of the actual RBC and the corresponding surface area. To ensure steady state before diamide addition, we also simulated the values of several antioxidant pathway related metabolites in the absence of diamide addition (S1 Text).

We simulated the response of healthy and G6PD deficient RBCs at 30% hematocrit after treatment with 0.25 mM diamide at t = 0 min, to fit the conditions of the previous experimental study [3]. G6PD catalyzes the first reaction of the pentose phosphate pathway, and is a key enzyme that helps maintain high levels of antioxidant metabolites such as GSH and reduced nicotinamide adenine dinucleotide phosphate (NADPH) in the cell. Therefore G6PD deficient RBCs have been known to have lower levels of these metabolites and thus are more susceptible to maintain homeostatic levels of these compounds after oxidative treatment [46]. The patient-specific parameters are given in Table 2 [47]. In addition to these conditions, we also traced clustered band 3 levels of the control RBC model during treatment with pulses of diamide with varying intervals (from 1 to 60 min), and concentrations (from 0 to 0.25 mM), to assess the properties of clustering under repeated oxidation.

**Table 2:** Model parameters for the healthy and G6PD deficient RBC (from [47]).

	V_max_ ((M/s)	K_mG6P_ (μM)	K_mNADP_ (μM)	K_iNADPH_ (μM)	K_iATP_ (μM)	K_i23BPG_ (μM)
Control	64	67	3.7	3.1	749	2289
G6PD^-^	1.1	152	3.8	0.62	180	520

### Extended model with effects of spectrin cytoskeleton

To study the interaction of the RBC membrane cytoskeletal network and its role in band 3 clustering, the above model was further extended to include compartments for positioning of spectrin and spectrin-bound band 3 molecules (S1A Fig). The model structure is explained in detail in S1 Text. In the extended model, the spectrin molecules were placed in contiguous rows to represent filaments. The filaments would intersect to form a group of equilateral triangles, and the spectrin-bound band 3 molecules were located at the intersection points. Such six-fold triangular structure has been used previously in several studies to represent the RBC spectrin cytoskeleton [48–51]. The edge lengths were set to 100 nm, as in the actual RBC membrane. Initial band 3 were defined as two different species; spectrin-bound band 3 which localizes at the intersection of spectrin filaments (BoundBand3), and free band 3 which has slightly confined diffusion compared to our non-spectrin model because of the physical spectrin barriers. The reaction schemes and rates for the oxidation induced changes in band 3 were left the same. To validate model accuracy, the position of a single freely diffusing molecule was tracked every 0.22 ms for 700 ms, and compared to the diffusional behavior of band 3 undergoing confined diffusion between compartments from previous experiments [57]. Furthermore, simulation of 0.25 mM diamide treatment in RBCs with different amounts of initial spectrin (S2 Table) was carried out to assess the effects of cytoskeletal defect on band 3 clustering.

### Reaction and diffusion properties of band 3

Spatiocyte performs diffusion of molecules at predefined intervals, computed from the voxel radius and the diffusion coefficient. The deterministic and stochastic reactions, however, are executed with event-driven step intervals. Phosphorylated band 3 molecules require an interval of 12 μs before walking to a neighbor voxel, whereas unphosphorylated band 3 molecules diffused much slower with an interval of 1.2 ms. For all reactions in the RBC model that does not consider the spectrin cytoskeleton (control model), we calculated the average interval for a reaction event to take place during steady state cluster formation (from t = 1000 s to t = 2000 s). Clustering of phosphorylated band 3 molecules, which is a diffusion-limited reaction, was the fastest, with an average interval of 337 μs. The slowest diffusion-influenced reaction was the dimerization of hemiBand3phos, which nucleates the hemiBand3cluster, at 400 ms on average. The fastest Gillespie’s next reaction was the first-order reaction that reduces Band3cluster to Band3phos, each event taking place at 0.7 ms intervals. The dephosphorylation of hemiBand3phos to hemiBand3oxi was the slowest stochastic reaction, with an average interval of 152 s. The fastest ODE reaction was S-glutathionylation that takes place every 39 ms, whereas G6PDH was the slowest deterministic reaction, with an average interval of 104 s.

For all diffusion-limited reactions in the model, the highest probability for a bimolecular reaction to occur upon collision is unity (Table 1). Therefore, the situation where the molecules stop diffusing until a reaction event takes place, does not arise. In all diffusion-influenced reactions, the diffusion step interval is shorter than the average reaction interval. For example, in the case of phosphorylated band 3 species, the diffusion interval is 12 μs whereas its shortest reaction interval is 337 μs (Band3phos + Band3cluster → Band3cluster + Band3cluster). As a result, the Band3phos molecule performs random walk on average 337/12 = 28 times before it collides with Band3cluster and reacts with unit probability. We use the modified Gillespie's next reaction to perform reactions involving a diffusing species such as band 3 and a chemical species such as diamide. In Diamide + Band3 → Band3oxi for example, when the reaction time is up, a single band 3 molecule is selected randomly and is converted to band3oxi while the diamide molecule number is decremented.

To evaluate the diffusion behavior of band 3 molecules, we measured the effective diffusion coefficient of unphosphorylated band 3 molecules under various conditions. We first diffused 100 band 3 molecules devoid of reactions and spectrin on a membrane with periodic boundary condition at the edges. We measured an average diffusion coefficient of 1.09×10^-14^ m^2^s^-1^, which agrees well with the specified diffusion coefficient (1.0×10^-14^ m^2^s^-1^) in the model. Increasing the number of molecules to 4800, which is the total number of band 3 in the control model, resulted in 8.1×10^-15^ m^2^s^-1^. This slowdown in diffusion is attributed to the excluded volume effect brought by the crowded band 3 molecules on the membrane. Next, we added all of the reactions in the control model but removed diamide to prevent clustering. We obtained the same effective diffusion coefficient of 8.1×10^-15^ m^2^s^-1^, which demonstrates that the reactions do not affect the diffusion behavior. Adding diamide to the model generated band 3 clustering and resulted with an effective diffusion of 7.2×10^-15^ m^2^s^-1^ during steady-state clustering (from t = 1000 s to t = 2000 s). However, measuring only the freely diffusing band 3 molecules gave the same coefficient of 8.1×10^-15^ m^2^s^-1^. Therefore, the slower diffusion coefficient during clustering is attributed to the fixed band 3 molecules in the clusters. Finally, when spectrin cytoskeleton was added to the model, its cage-like effect caused the diffusion coefficient of band 3 to drop drastically to 6.0×10^-16^ m^2^s^-1^, when measured for 100 s.

### Experimental methods

To study band 3 spatial distribution during diamide exposure, we utilized a technique that labels band 3 via the lysine-430 of their cytoplasmic domain [53]. Mouse RBCs were washed in 5% PBS glucose solution with 1 mM EDTA solution for preparation. For the diamide samples, RBCs were incubated in 0.125 mM diamide (from Sigma, St. Lewis, Missouri, USA) for 30 minutes at 37°C. Control and diamide-treated RBCs were then fixed using 4% paraformaldehyde, labeled with 0.5 mM eosin-5-maleimide (EMA; from Molecular Probes, Eugene, Oregon USA), and washed three times with PBS. Images were collected using an inverted microscope (Ti-U, Nikon) with a 100x 1.49 NA oil-immersion objective lens, an EMCCD camera with 512x512-pixel chip (iXon3, Andor Technology, Belfast, UK) and a fiber-coupled 488 nm laser. Eosin was excited by a 488 nm laser and fluorescence was collected through a 525/45 nm bandpass filter. NIS elements software (Nikon, Tokyo, Japan) was used for image acquisition and ImageJ software was used for image processing and final figure preparation.

## Results

### A particle model of oxidation induced spatio-temporal changes in RBC band 3

**Fig 3:**
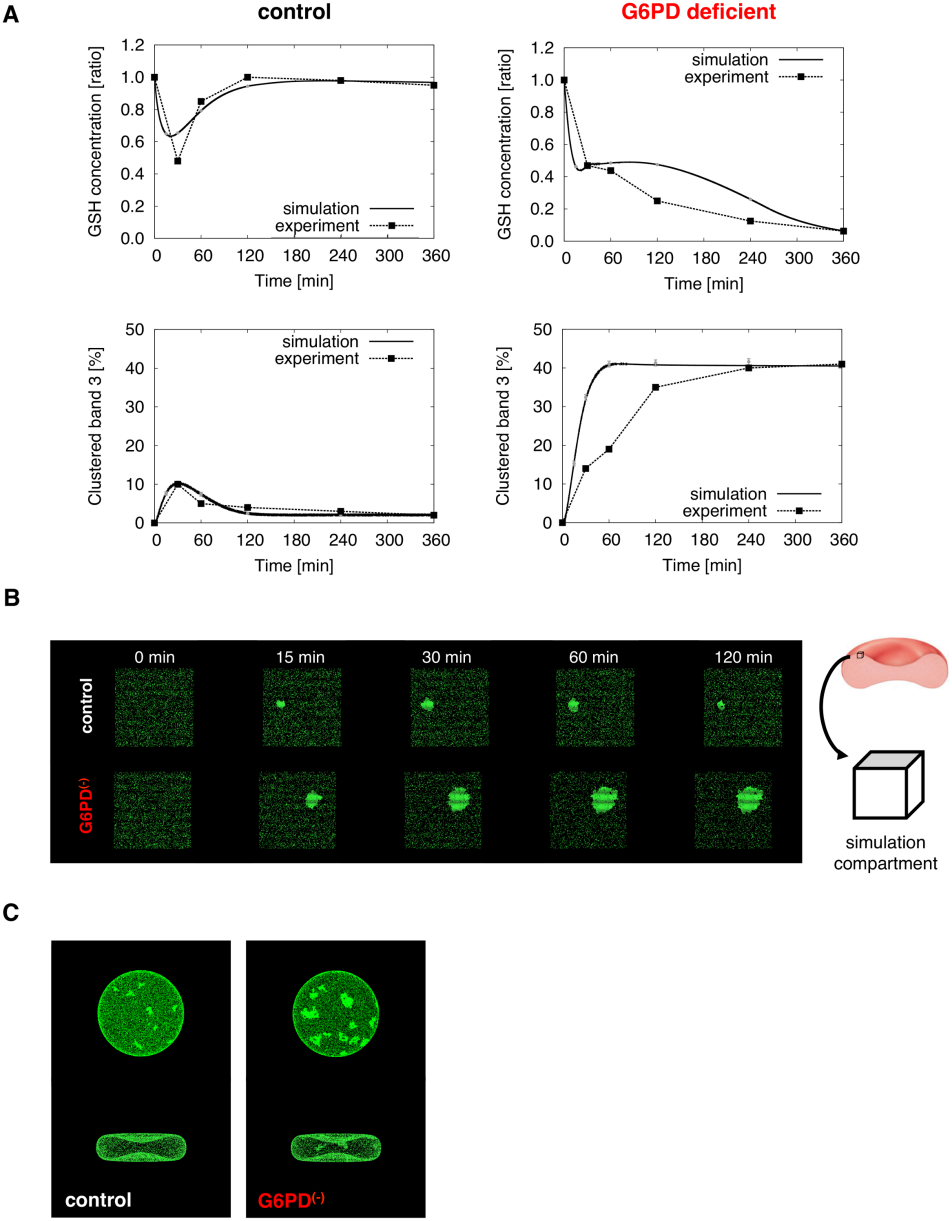
Measured and predicted biochemical changes in diamide treated RBCs. (A) Comparison between measured levels of GSH (top) and clustered band 3 (bottom) published by Pantaleo et al. ([3]) and corresponding simulated values, in healthy (left) and G6PD deficient (right) RBCs during diamide treatment. All simulations were performed using E-Cell System Environment Version 3.2.3pre2 installed with Spatiocyte, and the average for stochastic simulations of 100 runs was computed. (B) Visualization of simulation results showing inhomogeneous distribution of band 3. Each molecule represents a single band 3 dimer. The membrane is represented by the surface of a cuboid shape compartment with one-thousandth actual RBC volume. Error bars (indicated in grey) represent the standard deviation. The mean for the percentage of clustered molecules at t = 15, 30, 60, and 120 min are 7.61 (standard deviation, SD: 0.44), 10.2 (SD: 0.52), 7.42 (SD: 0.48), and 2.47 (SD: 0.25) for the control, and 15.3 (SD: 0.59), 32.4 (SD: 0.72), 41.0 (SD: 0.75), and 41.3 (SD: 0.77) for the G6PD deficient RBC, respectively. (C) Visual results of simulation on biconcave shape compartment one-hundredth actual RBC size (t = 30 min).

To better understand the mechanics of band 3 clustering, we developed a model that represents the changes in metabolites and band 3 after diamide treatment. When we treated RBC with 0.125 μM diamide for 30 min, the rate of cells exhibiting bright puncta was significantly increased compared with control condition (Fig 2A and S2 Fig), indicating that oxidation stress by diamide induces the rearrangement of band 3 in RBCs. The experimental result showed good agreement with previously published experimental data [3]. Our simulation results also produced similar patterns to our microscopy images. As illustrated in Fig 3A, transient decrease of GSH and increase of clustered band 3 levels were observed in the control RBC following diamide treatment. In addition, progressive and irreversible decrease of GSH and increase of clustered band 3 levels were observed in GP6D deficient RBC.

In the visuals (Fig 3B), relatively larger clusters were observed in the G6PD deficient RBC, compared to those of the control RBC. The mean for the percentage of clustered molecules at t = 15, 30, 60, and 120 min for 100 simulation runs was 7.61%, 10.2%, 7.42%, and 2.47% for the control, and 15.3%, 32.4%, 41.0%, and 41.3% for the G6PD deficient RBC, respectively. The differences in cluster size and distribution in our biconcave shape model results, were similar to a previous comparative imaging study of control and oxidant susceptible RBCs with visceral lesihmaniasis, (Fig 3C; [24]). Furthermore, clusters displayed growth until 30 min and then exhibited shrinkage in the control RBC, whereas clusters grew continuously in G6PD deficient RBCs.

### Affinity between clustered molecules shape cluster formation

**Fig 4:**
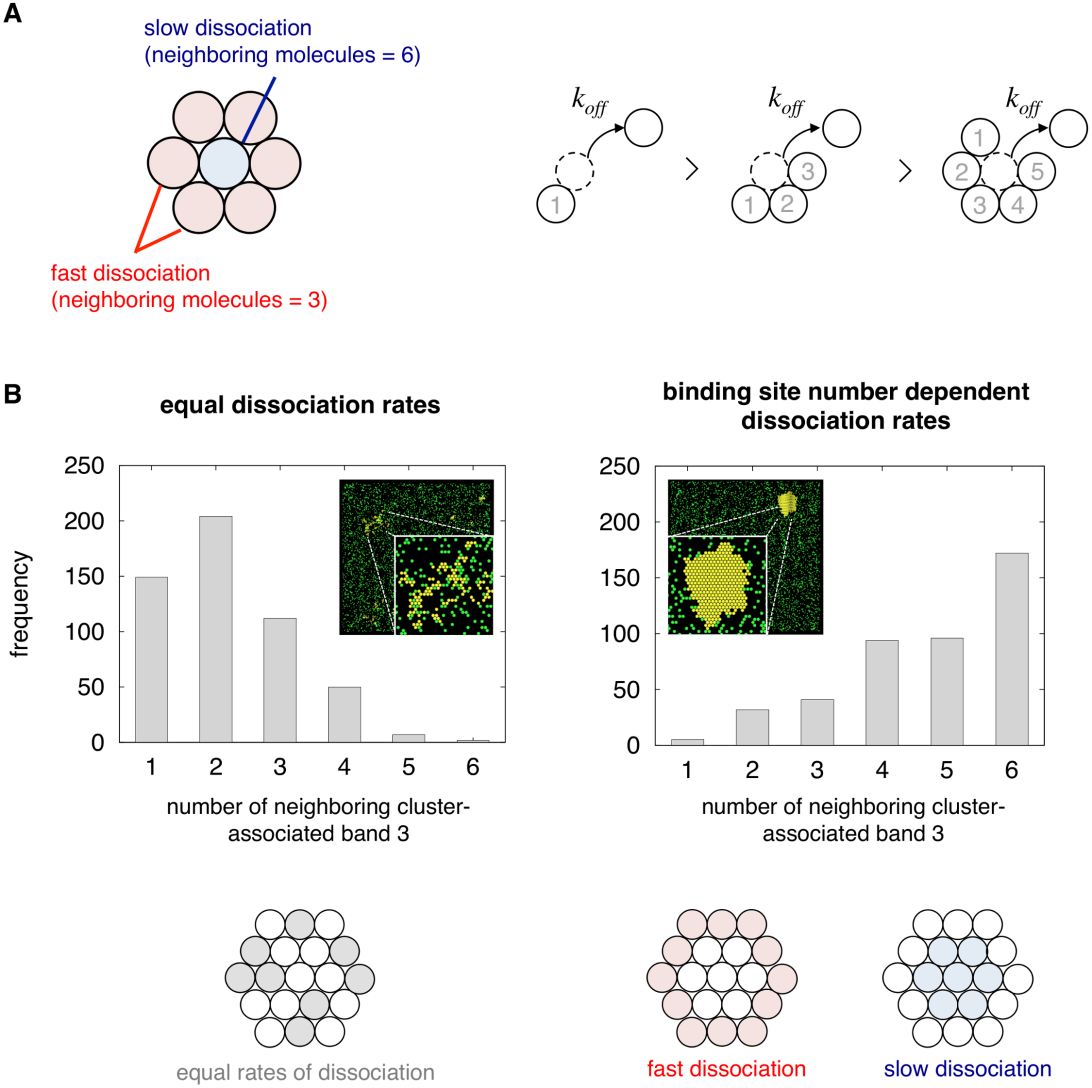
Effect of cluster dissociation reaction scheme on cluster morphology. (A) Scheme of cluster disassembly in the developed model. In the Spatiocyte model, space is discretized into a hexagonal close-packed lattice, with each surface voxel having 6 adjoining neighbors. Here we modified the model to have binding site number dependent dissociation rates, thus allowing molecules bound to fewer molecules, to dissociate from the cluster faster than those bound to many molecules. (B) Histograms of the number of neighboring cluster-associated band 3 for reaction scheme with equal rates for cluster dissociation (left) and binding site number dependent dissociation rates (right) at t = 30 min. Insets represent the visual output of the simulation, and close-ups of the clustered regions. Cluster associated band 3 are represented in yellow, remaining band 3 species are represented in green. In this analysis, a large number of molecules with a large number of neighbors represent a large cluster with densely packed molecules.

To determine the reactions that contribute to cluster formation, we assessed the influence of discarding several model specifications we had incorporated in our developed model. For example, we found that modifying the reaction settings for cluster dissociation rates resulted in greatly different morphological characteristics, even under the same simulation conditions of 0.25 mM diamide treatment. In our developed model, we had modeled cluster breakdown so that the dissociation rate of cluster-associated band 3 exponentially decreases with the addition of a neighboring molecule (Fig 4A, [54]). Hence, the more bound sites there are, the harder it is for the molecule to dissociate from the cluster. This bound-site dependent dissociation rate scheme always resulted in the formation of large, dense and stable clusters, closely representative of experimentally reported protein clusters (Fig 4B right). However, when representing this process with a first order reaction, where clustered band 3 is converted back to its freely phosphorylated state independent of its position, several sparse, static clusters were formed (Fig 4B, left). The difference was also quantified by comparing the number of bound sites of each clustered molecule in the two models (Fig 4B). At 30 min, the peak timing for clustered levels, a large population of molecules were bound to 1 to 3 molecules, whereas in the latter model, most were bound to 4 to 6 molecules. Therefore it could be implicated that affinity between clustered molecules is closely correlated with cluster shape formation dynamics.

### Irreversible hemichrome reactions contribute to cluster accumulation

**Fig 5:**
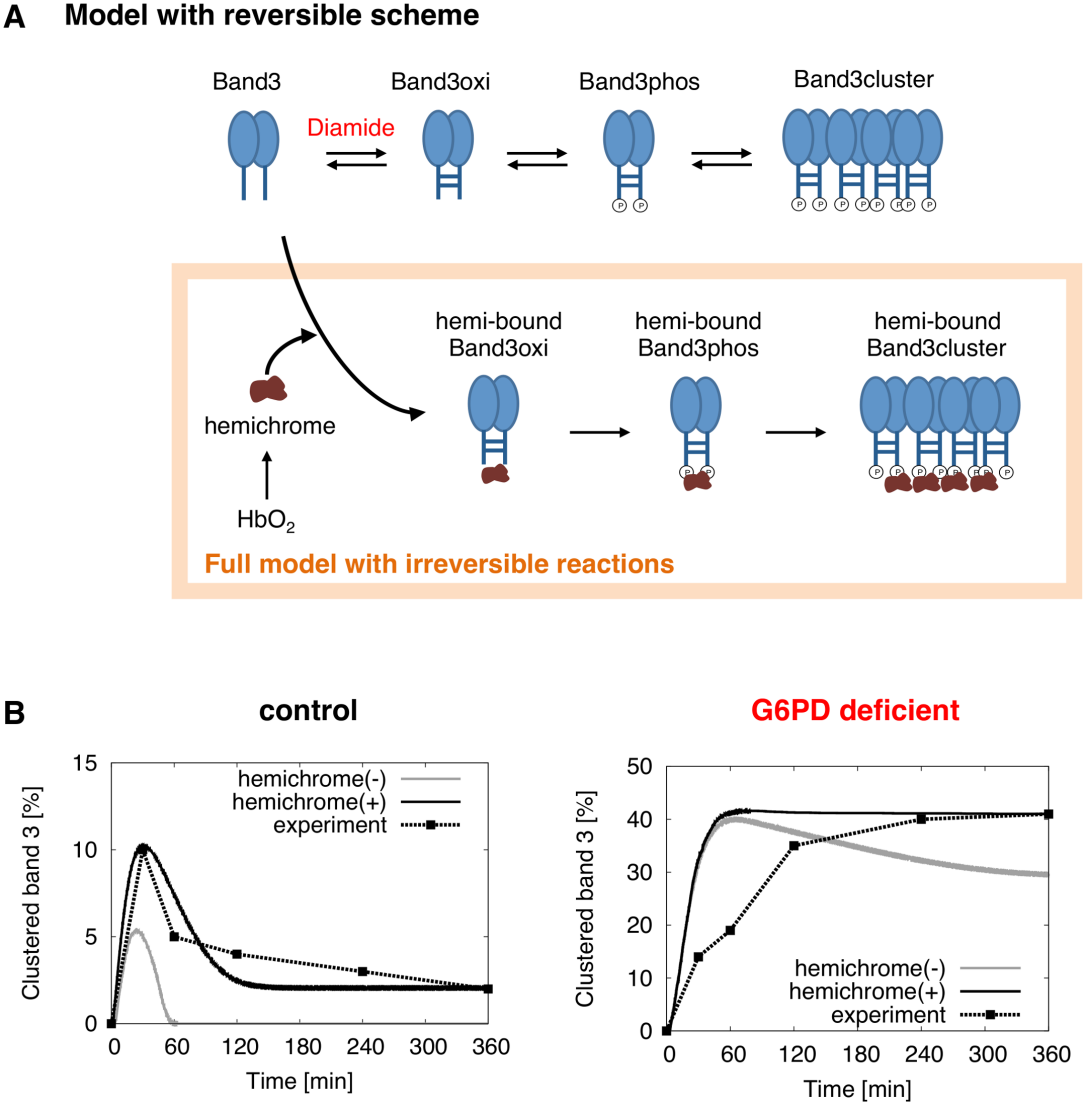
Addition of an irreversible hemichrome attachment scheme prolongs cluster lifetimes. (A) Schematic representation of a scheme where band 3 only reversibly undergoes transitions into oxidized, phosphorylated, and clustered state (top), and the full scheme of the developed model (bottom) including irreversible formation and attachment of denatured hemoglobin, known as hemichrome, to band 3. (B) Comparison of predicted clustered band 3 levels in using the scheme with only reversible reactions (grey), the developed model (solid black), and experimental results (dotted black, [3]) for control and G6PD deficient RBCs. The graphs for the developed model and experimental results are the same as those of Fig 3, but have been reprinted for comparison.

In developing our model, we attempted to reproduce two main properties of band 3 clustering in control RBCs observed from the experimental data [3]; the transient clustering of band 3, and the maintenance of low levels of clustered band 3 over a long period following diamide exposure. During this process, we found that cluster reversibility is greatly affected by the presence of irreversible state transitions of band 3. When representing the sequential reactions leading to cluster formation with only a fully reversible scheme (Fig 5A, top), we found that a high rate of reverse reactions (reduction, dephosphorylation, and cluster dispersion) compared to forward rates, was required to reproduce the transient behavior (Table 1). However, the high rate also resulted in the complete breakdown of the cluster at t = 60 min in the control RBC (Fig 5B). As this contradicted the second property, we speculated that a portion of the clustered band 3 undergo irreversible transition into a state that inhibits its breakdown.

A number of previous studies have pointed out the role of hemichrome, a product of hemoglobin (Hb) denaturation, in promoting band 3 clustering [19,55,56]. Hb is oxidized by molecular oxygen to form ferric methemoglobin (metHb). Although metHb can be reduced back to Hb by a nicotinamide adenine dinucleotide (NADH)-dependent reducing system, it has been shown that the oxidation process can be followed by the transformation of metHb into hemichrome. The hemichrome then binds to the cytoplasmic domain of band 3, forming an insoluble copolymer, which is suggested to play a key role in the control of damaged and aged cells in the blood circulation.

In the developed model, we added a reaction where hemichrome formed by strong autoxidation is attached to cluster-associated band 3 molecules to disable them from returning to their original freely diffusing state (Fig 5A; Table 1 equation 9–15). As a result, clustered levels remained present even after 60 min (Fig 5B). Similarly, whereas without the hemichrome formation reaction G6PD deficient RBCs exhibited transient clustering of band 3 molecules, the addition of the reaction resulted in formation of permanent clusters (Fig 5B). These results suggest that oxidation induces formation of hemichrome associated clusters, which extend cluster lifespan and prolong its effects, even hours after the oxidative insult.

### Control RBCs exhibit progressive band 3 clustering under repeated oxidation

**Fig 6:**
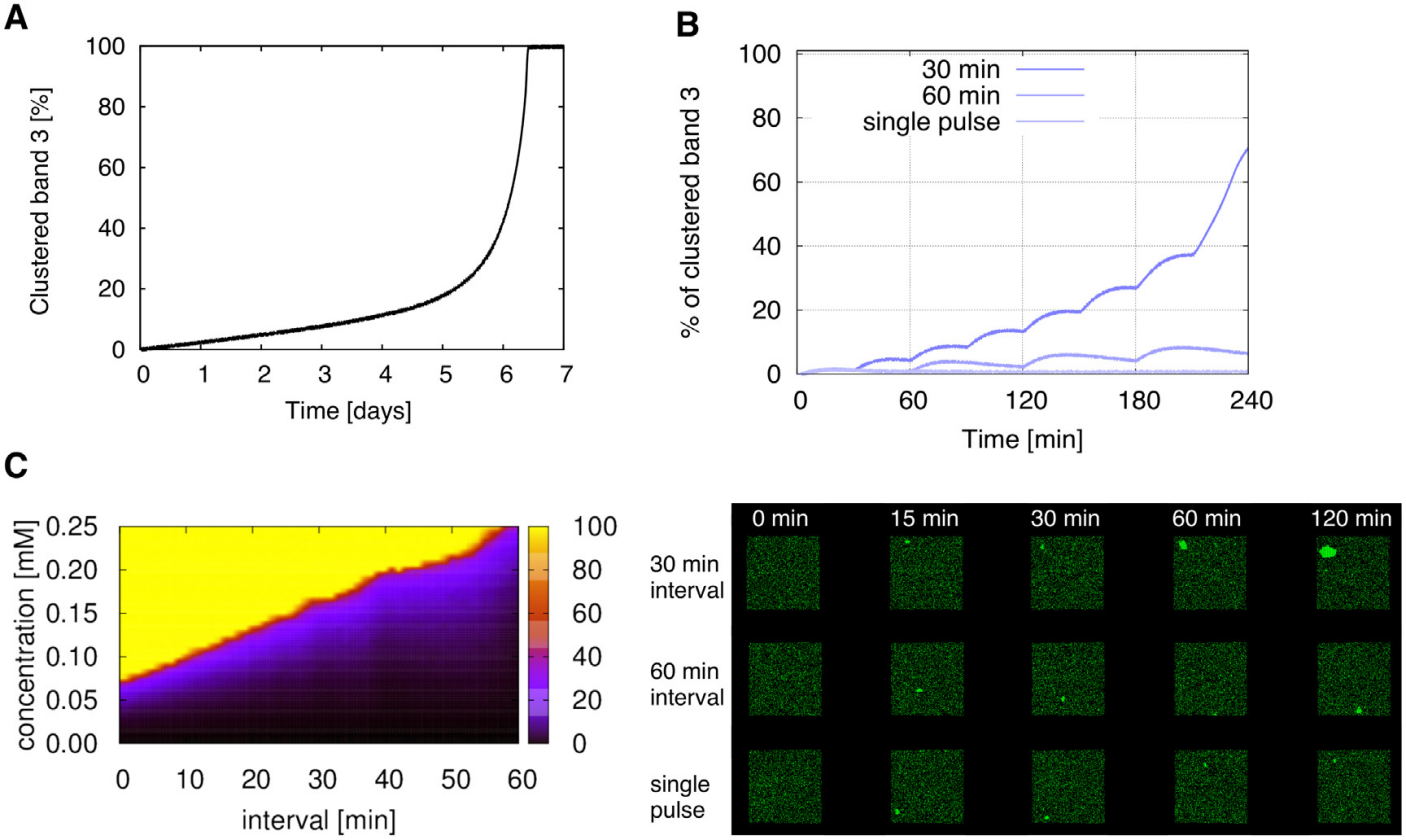
Repeated oxidation triggers irreversible clustering in healthy RBCs. (A) Clustered band 3 levels during addition of 0.25 μM diamide at 10s intervals for 1 week. (B) Clustered band 3 levels during single pulse and repeated perturbations by 0.125 mM diamide at 30 and 60 min intervals (left), and corresponding visual output (right). (C) Heatmap of clustered band 3 levels with the x-axis as pulse intervals, and y-axis as diamide concentration, and z-axis as percentage of clustered band 3 at t = 60 min.

Human RBCs have a lifespan of 120 days, during which they are continuously exposed to small amounts of oxidative stress from superoxides and hydrogen peroxide [52]. Since previous experiments have only assessed band 3 clustering after a single oxidative event, we applied the model to observe the consequences of multiple oxidative perturbations, to mimic a more physiological situation of oxidative stress. Dosages of 0.25 μM diamide were added to the control RBC model at 10 second intervals, and clustered band 3 levels were traced for seven days. Interestingly, although previously cluster formation was shown be reversible in control RBCs, irreversible gradual increase of clustered band 3 was observed for the first few days, followed by an exponential increase and saturation of levels after day 6 (Fig 6A).

To closely investigate the elevation of clustered levels, we ran simulations with longer time intervals (30 min and 60 min) and a higher concentration of 0.125 mM. For both simulations, transient clustering was accompanied with each pulse, and the basal level of the clustered band 3 was increased over time (Fig 6B). In the 60 min simulation, the clustered levels did not decline after approximately t = 200 min, resulting in irreversible cluster growth. Diamide pulses appeared to enhance cluster growth, rather than the increase of cluster number.

Analysis of the clustering behavior with varying diamide concentrations and addition intervals, showed that addition of higher concentration of diamide with shorter intervals lead to rapid saturation of clustered levels. At t = 120 min approximately 44.3% of the simulation conditions resulted in the saturation of clustered band 3 levels (100% clustered levels), and 35.2% of them resulted in clustered levels of less than 10% of total band 3 (Fig 6C). Saturation of clustered levels became more apparent as time proceeded (S3 Fig), however the portion of conditions that resulted in 40 to 80% of band 3 to become clustered did not change over time.

### Simulation of oxidation in model incorporating spectrin interactions

**Fig 7:**
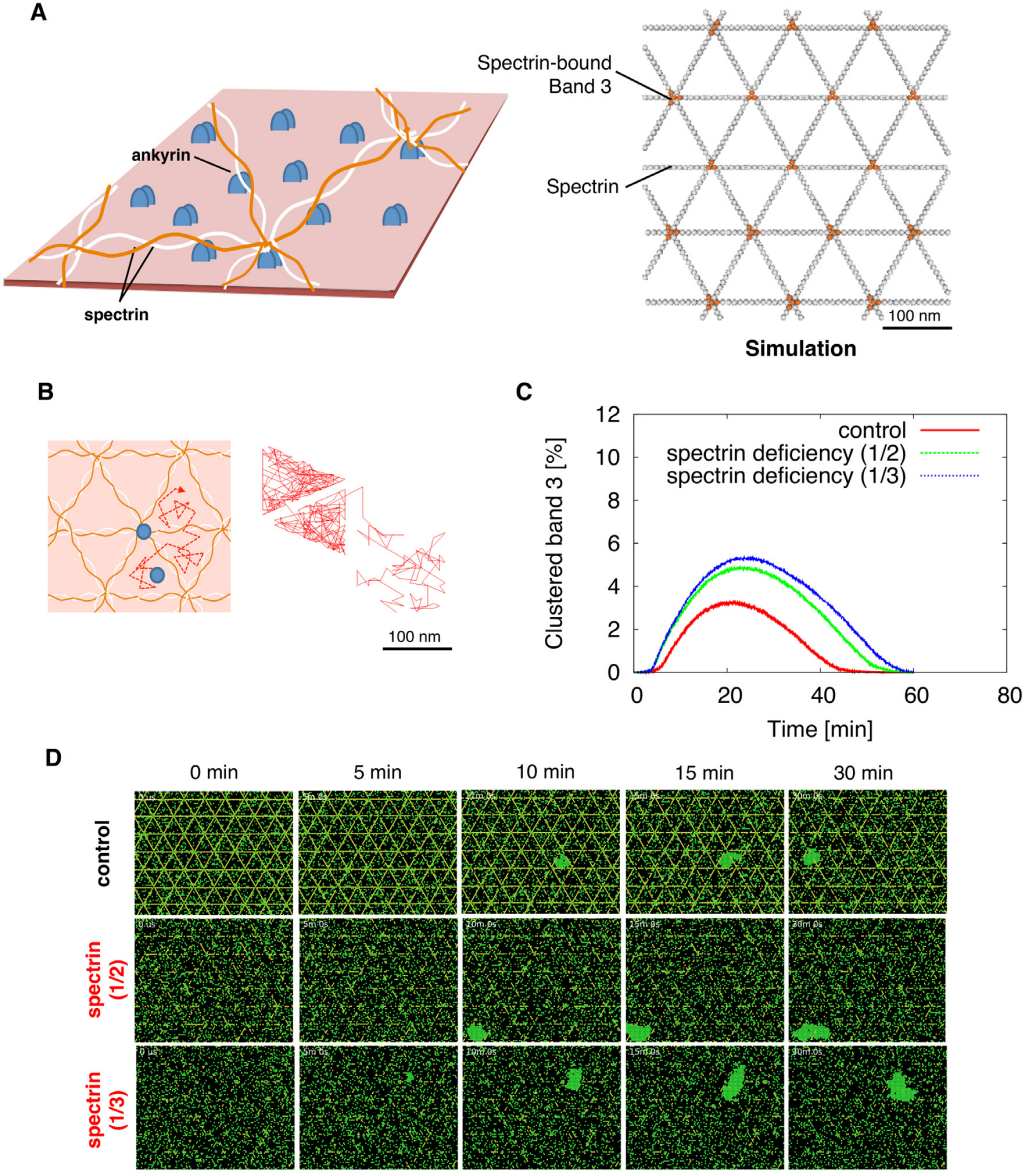
Spectrin deficiency enhances band 3 cluster formation in the spectrin network compartment model. (A) The erythrocyte membrane skeleton is organized as a polygonal network formed by spectrin molecules linked to ankyrin, which is bound to the cytoplasmic domain of band 3 (right). This spectrin-based RBC membrane cytoskeletal network was reproduced in Spatiocyte (right) where light grey molecules represent spectrin, and orange molecules represent spectrin bound band 3. (B) A typical simulated trajectory of band 3 undergoing confined diffusion observed with a time resolution of 0.22 ms for total observation time of 700 ms. (C) Comparison of the clustered band 3 levels in the spectrin network band 3 membrane model, for control (red), half of spectrin present (green), and one third of spectrin present (blue). (D) Corresponding visual output of simulation results. Spectrin and band 3 molecules are shown in yellow and green, respectively.

At normal state, the RBC membrane cytoskeleton takes the form of a hexagonal lattice, which is held together by junctions where spectrin and other membrane-spanning proteins such as band 3 interact [58]. Thus, spectrin is suggested to play an important role in regulating the diffusion of band 3. In our original model we had allowed band 3 to be homogeneously distributed during initialization, so here we extended the original model by including additional RBC membrane components and related reactions (Fig 7A; S1 Text).

A number of prior studies have shown that band 3 which are not attached to the cytoskeleton exhibit a “hop-diffusion” like movement, where they diffuse freely within the spectrin barriers, and occasionally when the spectrin tetramers transiently dissociate thereby creating a gap in the “fence”, they are able to diffuse to a separate neighboring compartment [52,59,60]. To reproduce this, we simulated and compared the trajectory of free band 3 in normal state with spectrin present, with the trajectories obtained from a prior experimental study [52]. The locus of diffusing band 3 molecules simulated for 200 sec (example shown in Fig 7B) was similar to those previously observed in the experiment. Simulation of the temporal position (coordinates) predicted that band 3 hops to an adjacent mesh at an average of every 347.2 ms. This was in good agreement with the experimentally obtained residency times for each domain, with an average of 350 ms [52].

Finally, as an application of our band 3 clustering spectrin model, we predicted the behavior of clustering in RBCs with a membrane cytoskeletal disorder, namely spectrin deficiency. In the RBC, the spectrin-based membrane skeleton is responsible for the unique flexibility and mechanical stability of the cell. Spectrin defect is known to result in the loss of membrane stability, leading to surface area loss and hereditary spherocytosisa, a common hemolytic anemia characterized by the production of irregular sphere-shaped RBCs [61]. From our simulations, we found that band 3 clustering levels were more pronounced in the spectrin deficient RBC models by roughly two-folds (Fig 7C). Also, clusters of larger size were observed in the spectrin deficient RBCs, whereas in the controls, cluster size was partially confined within the spectrin filament physical barriers (Fig 7D).

## Discussion

In this study, we developed a computational model of biochemical changes in human RBCs to study band 3 distribution during oxidative treatment. We integrated the cytoplasmic biochemical reactions and diffusion-influenced reactions on the RBC membrane and parameterized the model using time-course data and fluorescent images from previous works. From the model, we were able to speculate the factors that contribute to the remodeling of band 3 clustering behavior observed in previous experimental studies, and predict how cluster formation is affected by the conditions that the cell is put under.

It has long been discussed that oxidation induced clustering of band 3 is an essential process in RBC senescence. Although experimental approaches have described the macroscopic effects on the RBC during oxidative treatment, the properties of cluster formation and how it behaves under various conditions have remained limited. Specifically, questions such as what causes the irreversibility of clustering in G6PD deficient cells, how are band 3 clusters formed *in vivo* during aging, and how does the physical architecture of the membrane contributes to cluster formation, are yet to be answered.

Prior experimental and computational studies of the changes in RBC metabolism during oxidative treatment have suggested a decline in essential antioxidant metabolites such as GSH and NADP, and in turn an increase in GSSG and reduced nicotinamide adenine dinucleotide phosphate (NADPH), following oxidation. As reduced levels of antioxidants have been observed in RBCs with disorders associated with short RBC lifespan, using these models it is possible to predict the consequences of oxidative stress on RBC function and lifespan, to a certain degree. However, several reports showing that the decrease of enzyme activity and metabolite levels are nonlinear with cell age [21,62], suggest that metabolites levels alone are not sufficient to allow the direct assessment of RBC health. Our work, for the first time focuses on modeling beyond the changes in metabolism, hence the effects of oxidation on the RBC membrane. Since the clustering of band 3 proteins induced by oxidative stress is a process that directly promotes cell removal, our work enables a more detailed and visual description of the oxidative events that occur during the RBC life cycle.

The simulations of diamide treatment with our models were consistent with the reported characteristics of the cells *in vitro*. The levels of clustered band 3 rose to a peak at about 30 min, and then declined in the control, and progressively increased in the G6PD deficient RBC (Fig 3A). Visualization of the simulation results also showed good agreement to the previously described band 3 clusters (Fig 3B and 3C; [3,22–25]), and revealed larger clusters in the G6PD deficient cell compared to the control. The fact that the clusters are greater in size, rather than in abundance, is consistent with prior studies that have observed clusters with larger diameters in RBCs with disorders [23,24]. As it has been shown that increased oxidative stress exposure in G6PD deficient patients can induce the premature destruction (or hemolysis) of up to 25–30% of RBCs within hours [63], we can speculate that the formation of large clusters is a significant step that accelerates the removal process of RBCs damaged from oxidation.

In the course of model development, we found that the number of clusters and their morphological characteristics are greatly influenced by the ratio between cluster nucleation rate and cluster growth rate (S4 Fig). Parameterizing our model so that the cluster size and number of clustered associated molecules matched the previously reported values [23], we predicted that cluster nucleation rate is slower than the cluster growth rate (Table 1 equations 6, 7), thus it is easier for diffusing phosphorylated molecules to bind to existing clusters than newly forming another. Also, it was found that implementing binding site number dependent dissociation rates for clustered molecules leads to formation of densely packed clusters (Fig 4A and 4B). This property also resulted in the clusters to appear highly dynamic, with relatively high exchange of molecules on the cluster exterior, and long residence time of molecules on the cluster interior (Fig 4B). This has been observed in previous studies as a critical feature of membrane protein clusters [26,64]. These results together indicate that strong affinity between clustered molecules play an important role in the clustering of band 3 in RBCs.

Fitting the model to the experimentally derived band 3 cluster curves in control RBC, we observed that band 3 has a high tendency to return to its natural unclustered state. This is supported by prior experimental evidence of highly active protein tyrosine phosphatases resulting in low basal levels of phosphorylated band 3 [65], and high antioxidant defense systems present in RBCs. Testing a variety of reaction schemes, we also found that in the absence of an irreversible reaction which inhibits cluster dispersion, the model failed to reproduce the prolonged levels of clustered band 3 reported in previous studies [3; Fig 5A and 5B]. Including additional pathways that describe the irreversible binding of hemichrome to band 3 of different states resulted in closer representation of experimental results. Although some studies have not identified hemichrome formation in healthy RBCs [3], other studies have shown that oxyHb is highly susceptible to hemichrome formation even under physiological temperature and pH [63]. Additionally, high levels of hemichromes bound to band 3 clusters have been observed in pathological RBCs associated with hemolysis [3,55], and it has also been shown that direct removal of hemichromes largely decreased the number of clusters [20]. From such analyses we hypothesize that small amounts of hemichrome act as a glue-like substance that strengthens the cluster making it difficult for the clustered band 3 to return to its natural, diffusing state.

Applications of our developed model led to several biological predictions. First, our simulations predicted that repeated perturbations of diamide could trigger irreversible growth of band 3 clusters even in control RBCs (Fig 6). While it has been previously reported that clusters in healthy RBCs represent different clustering characteristics than in G6PD deficient RBCs when treated with diamide [3], here we found similar progressive clustering behavior when the healthy RBCs were repeatedly treated with short intervals. The tendency of phosphorylated band 3 to bind to pre-existing clusters likely promotes this process, enlarging clusters with each pulse, in a positive feedback like manner. From this we can consider that although clustering is generally a transient process in healthy cells, the accumulation of oxidative damage leads to irreversible clustering, eventually resulting in cell removal.

Cluster growth showed to be largely dependent on the interval between diamide exposure times. Even under small concentrations such as 0.25 μM, treatment for 10 second intervals for a long period of time resulted in an exponential increase of clustered levels (Fig 6A). Similar nonlinear changes in GSH levels and membrane bound globin have been observed in dog RBCs *in vivo* [66]. Additionally, a generated heatmap of clustered levels under various conditions of multiple diamide exposure, represented distinctive regions rather than a gradual gradient, suggesting that most conditions result in either low clustered levels or high saturation levels of > 90%. The reason for this trend of clustered levels falling into two distinctive states has yet to be evaluated, however it could be closely related to simplifying the process of easily telling apart the cells that are ready to be removed from the circulation, from those that are not. Such process may be relatively difficult if there exists many cells containing clustered band 3 levels spread across a wide range.

Recent studies have addressed the important contribution of membrane partitioning on the biomolecular reactions that occur inside the cell [67,68]. As mentioned earlier, band 3 contributes significantly to maintaining the plasticity and integrity of the RBC membrane, through the attachment of a third of its population to cytoskeletal spectrin. In contrast to our primary model of homogenous initial distribution of band 3, we included membrane structural components and additional reactions in the spectrin model to assess how clustering would differ in RBCs with a cytoskeletal disorder. From our simulations it was found that diamide treatment had a greater effect on RBCs with spectrin deficiency (Fig 7D), producing clusters of larger size. Our results suggest the spectrin cytoskeleton functions as a physical barrier to suppress band 3 clustering at steady state.

It is important to note that there are several limitations to these models. In order to reproduce our system of interest under limited parameter and reaction data, we have focused on only the reactions associated with oxidation induced band 3 related changes, thus neglecting the known interactions between band 3 and other intracellular components. In the future, the model could be expanded to incorporate more detailed models of human RBC metabolism and band 3 related reactions, such as interactions between band 3, glycolytic enzymes, and hemoglobin, to assess the consequences of band 3 modifications on metabolism. Another limitation is that the mechanical effects of clustering, such as changes in membrane geometry are not included in the model. Future work is required to model the morphological changes of the membrane during band 3 clustering, and its feedback to the cell interior. Such extension may also help test hypotheses that will aid understanding of why RBCs dispose a large portion of their Hb, band 3, and membrane, during oxidative events through self-vesiculation, despite the membrane instability that it induces [69].

In conclusion, our work presents a particle simulation model that enables the prediction of how the distribution of band 3 is at any given time during oxidative treatment, and how it would be affected by the conditions that the cell is put under. If the amount of band 3 clustering can be predicted, this could lead to detection of methods to control this *in vitro* and *in vivo*. We expect our model to serve as a framework not only for understanding the molecular basis of senescent cell recognition by macrophages, but also to target drug-loaded RBCs to macrophages for specific therapeutic purposes, and optimization of blood storage, where clustering of band 3 is of relevance.

## Supporting Information

S1 Text Detailed description of the band 3 clustering model and its parameter settings. (PDF)

S1 Fig Comparison of band 3 clustering in the original model and the model with a vacant underlying spectrin network. (A) Visualization of model structure in the original model with homogenously distributed Band 3, and model with spectrin, spectrin-bound Band 3 (BoundBand3), and corresponding vacant compartment species (edge and vertex). (B) Comparison of visual output at initial state (left), and levels of clustered band 3 (right) in the original band 3 model, and spectrin model with same reactions but using vertex and edge compartments.
(TIF)

S2 Fig Comparison of band 3 distribution in control and diamide treated cells. The x-axis represents the number of cells with inhomogeneous fluorescence distribution divided by the total number of cells. Cells were counted in 10–15 fields and data was obtained from >10 cells in each field. The analyses of data are shown as mean±SD of three independent experiments. Statistical analysis was performed between the control and the diamide-treated cells (n > 10, **p < 0.01 by *t*-test).
(TIF)

S3 Fig Heatmap representation of clustered band 3 levels in RBC treated with indicated pulse intervals and diamide concentration at various times (in minutes). The x-axis represents the interval between pulses, y-axis represents the added diamide concentration, and z-axis represents the percentage of clustered band 3.
(TIF)

S4 Fig Cluster distribution and morphology under various rates of cluster nucleation and growth. Images of cluster formation at t = 30 min. The x-axis represents the probability of molecules forming a new cluster upon collision, and the y-axis represents the probability of molecules adding on to a pre-existing cluster.
(TIF)

S1 Table Model setting parameters for the initial band 3 clustering model. (XLSX)

S2 Table Model setting parameters for the spectrin network band 3 clustering model. (XLSX)
