# Supplementary material for: Particle Simulation of Oxidation Induced Band 3 Clustering in Human Erythrocytes
Source: PLoS Comput Biol. 2015 Jun 5;11(6):e1004210. doi: 10.1371/journal.pcbi.1004210 (PMC4457884; doi:10.1371/journal.pcbi.1004210)
Supplement: S1 Text — (PDF) [file pcbi.1004210.s001.pdf]

Supporting information (Shimo *et al.*)

Text S1.

1. Detailed description of human erythrocyte band 3 clustering model.

The main body of our band 3 clustering model is based on a previously published kinetic model of human erythrocyte (RBC) metabolism [1]. The minimal reactions associated with the antioxidant pathway shown below, were extracted from the original whole RBC metabolism model.

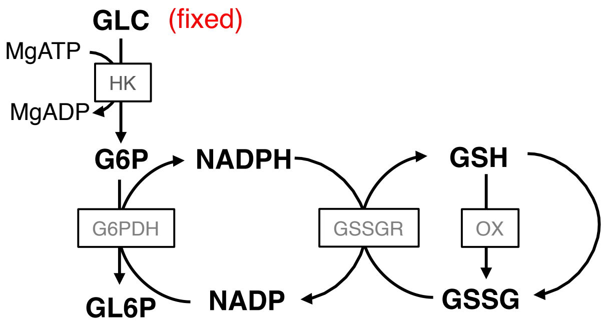

Figure 1. Schematic representation of metabolic reactions included in our model (minimal model)

1-1. Initial and steady-state concentrations of all substrates used in the model and details of the enzymatic reactions

Abbreviations of all reactions and reactants expressed in the developed minimal model are shown in Table 1.

Table 1: Steady-state concentrations of metabolic intermediates in the RBC model CYTOPLASM

Variable	Abbreviation	Concentration [mM]
Glucose	GLC	5.00E-03
Glucose 6-phosphate	G6P	6.00E-02
Gluconolactone 6-phosphate	GL6P	5.30E-06
Carbon dioxide	CO2	1.20E+00
Glutathione (reduced)	GSH	3.30E+00
Glutathione (oxidized)	GSSG	4.70E-03
Nicotinamide adenine phosphate	NADP	6.50E-05
Nicotinamide adenine phosphate	NADPH	6.50E-02
Free 2,3-Diphosphoglycerate	f23DPG	2.30E+00
Free adenosine diphosphate	MgADP	1.10E+01
Free adenosine triphosphate	MgATP	1.30E+01
Free guanosine diphosphate	GDP	9.60E-02
Free orthophosphate	Pi	1.00E+00

Below (Table 2) is a list of processes that structure the model.

Table 2: Enzymatic reactions included in the models

Enzyme / Process	Abbreviation	Substrates		Products
Hexokinase	HK	GLC + MgATP	→	G6P + MgADP
Glucose 6-phosphate dehydrogenase	G6PDH	G6P + NADP	→	GL6P + NAPDH
Glutathione reductase	GSSGR	GSSG + NADPH	→	GSH + MgADP + Pi
Glutathione turnover	OX	2GSH	→	GSSG

1-2. Kinetic equations and parameters of metabolic reactions used in the model.

Below we present a detailed description of the kinetic equations and parameters used in the model. The abbreviations of enzymes and metabolites correspond to those shown in Table 1.

HK

$$v=\frac{e_t\left(\dfrac{theK_{catf}\mathrm{AB}}{K_{i,\mathrm{B}}K_{m,\mathrm{A}}}-\dfrac{theK_{catr}\mathrm{PQ}}{K_{i,\mathrm{Q}}K_{m,\mathrm{P}}}\right)}{1+\dfrac{\mathrm{A}}{K_{i,\mathrm{A}}}+\dfrac{\mathrm{B}}{K_{i,\mathrm{B}}}+\dfrac{\mathrm{AB}}{K_{i,\mathrm{B}}K_{m,\mathrm{A}}}+\dfrac{\mathrm{P}}{K_{i,\mathrm{P}}}+\dfrac{\mathrm{Q}}{K_{i,\mathrm{Q}}}+\dfrac{\mathrm{PQ}}{K_{i,\mathrm{Q}}K_{m,\mathrm{Q}}}+\sum_{j=1}^{4}\dfrac{\mathrm{I}_j\mathrm{B}}{K'_{i,\mathrm{I}j}K_{i,\mathrm{B}}}} \quad \text{(S1)}$$

$$theK_{catf}=\frac{1.662k_{catf}}{\left(1+\dfrac{10^{-\mathrm{pH}}}{10^{-7.02}}+\dfrac{10^{-9.55}}{10^{-\mathrm{pH}}}\right)} \quad \text{(S2)}$$

$$theK_{catr}=\frac{1.662k_{catr}}{\left(1+\dfrac{10^{-\mathrm{pH}}}{10^{-7.02}}+\dfrac{10^{-9.55}}{10^{-\mathrm{pH}}}\right)} \quad \text{(S3)}$$

Symbols: A, MgATP; B, GLC; P, G6P; Q, MgADP; I, Pi, 2,3-BPG and GDP

Parameter	Value
e_t (M)	2.50E−08
$K_{m,\mathrm{MgADP}}$, $K_{i,\mathrm{MgADP}}$ (M)	1.00E−03
$K_{m,\mathrm{MgATP}}$, $K_{i,\mathrm{MgATP}}$ (M)	1.00E−03
$K'_{i,2,3\mathrm{BPG}}$ (M)	2.70E−03
$K'_{i,\mathrm{GSH}}$ (M)	3.00E−03
$K'_{i,\mathrm{GDP}}$ (M)	1.00E−05
$K'_{i,\mathrm{G6P}}$ (M)	1.00E−05
$K_{i,\mathrm{GLC}}$ (M)	4.70E−05
$K_{m,\mathrm{G6P}}$, $K_{i,\mathrm{G6P}}$ (M)	4.70E−05
k_{catf} (s^{-1})	180
k_{catr} (s^{-1})	1.16

Parameter values were taken from [2].

G6PDH

$$v=\frac{V_m\dfrac{\mathrm{AB}}{K_{m,\mathrm{A}}K_{m,\mathrm{B}}}}{1+\dfrac{\mathrm{B}}{K_{m,\mathrm{B}}}\left(1+\dfrac{\mathrm{A}}{K_{m,\mathrm{A}}}\right)+\dfrac{\mathrm{P}}{K_{m,\mathrm{P}}}+\dfrac{\mathrm{ATP}}{K_{\mathrm{ATP}}}+\dfrac{2{,}3\text{-BPG}}{K_{2,3\text{-BPG}}}} \quad \text{(S4)}$$

Symbols: A, G6P; B, NADP; P, NAPDH

Parameter	Value
K_{ATP} (M)	7.49E−04
$K_{\mathrm{2,3BPG}}$ (M)	2.29E−03
$K_{m,\mathrm{G6P}}$ (M)	6.67E−05
$K_{m,\mathrm{NADP}}$ (M)	3.67E−06
$K_{m,\mathrm{NADPH}}$ (M)	3.12E−06
V_m (M s^{-1})	6.40E-05

Parameter values were taken from [3].

GSSGR

$$v = \frac{e_t(N_1\mathrm{AB} - N_2\mathrm{P}^2\mathrm{Q})}{GSSGRrd} \quad \text{(S5)}$$

$$GSSGR_{rd} = D_1 + D_2\mathrm{A} + D_3\mathrm{B} + D_4\mathrm{P} + D_5\mathrm{Q} + D_6\mathrm{AB} + D_7\mathrm{AP} + D_8\mathrm{BQ} + D_9\mathrm{P}^2 + (D_{10} + D_{11})\mathrm{PQ} + (D_{12} + D_{13})\mathrm{ABP} + D_{14}\mathrm{AP}^2 + D_{15}\mathrm{BPQ} + D_{16}\mathrm{P}^2\mathrm{Q} + D_{17}\mathrm{ABP}^2 + D_{18}\mathrm{BP}^2\mathrm{Q} \quad \text{(S6)}$$

$$N_1 = k_1k_3k_5k_7k_9k_{11}$$
$$N_2 = k_2k_4k_6k_8k_{10}k_{12}$$
$$D_1 = k_2k_9k_{11}(k_4k_6 + k_4k_7 + k_5k_7)$$
$$D_2 = k_1k_9k_{11}(k_4k_6 + k_4k_7 + k_5k_7)$$
$$D_3 = k_3k_5k_7k_9k_{11}$$
$$D_4 = k_2k_4k_6k_8k_{11}$$
$$D_5 = k_2k_9k_{12}(k_4k_6 + k_4k_7 + k_5k_7)$$
$$D_6 = k_1k_3(k_5k_9k_{11} + k_6k_9k_{11} + k_7k_9k_{11} + k_5k_7k_9 + k_5k_7k_{11})$$
$$D_7 = k_1k_4k_6k_8k_{11}$$
$$D_8 = k_3k_5k_7k_9k_{12}$$
$$D_9 = k_2k_4k_6k_8k_{10}$$
$$D_{10} = k_2k_4k_6k_8k_{12}$$
$$D_{11} = k_2k_{10}k_{12}(k_4k_6 + k_4k_7 + k_5k_7)$$
$$D_{12} = k_1k_3k_8k_{11}(k_5 + k_6)$$
$$D_{13} = k_1k_3k_5k_7k_9k_{10}$$
$$D_{14} = k_1k_4k_6k_8k_{10}$$
$$D_{15} = k_3k_5k_7k_{10}k_{12}$$
$$D_{16} = k_8k_{10}k_{12}(k_2k_4 + k_2k_5 + k_2k_6 + k_4k_6)$$
$$D_{17} = k_1k_3k_8k_{10}(k_5 + k_6)$$
$$D_{18} = k_3k_8k_{10}k_{12}(k_5 + k_6)$$

Symbols: A, NADPH; B, GSSG;P, GSH; Q, NADP

Parameter	Value
e_t (M)	1.25E−07
k_1 (M^{-1} s^{-1})	8.50E+07
k_2 (s^{-1})	5.10E+02
k_3 (M^{-1} s^{-1})	1.00E+08
k_4 (M^{-1} s^{-1})	5.60E+05
k_5 (s^{-1})	8.10E+02
k_6 (s^{-1})	1.00E+03
k_7 (s^{-1})	1.00E+06
k_8 (M^{-1} s^{-1})	5.00E+07
k_9 (s^{-1})	1.00E+06
k_{10} (M^{-1} s^{-1})	5.00E+07
k_{11} (s^{-1})	7.00E+03
k_{12} (M^{-1} s^{-1})	1.00E+08

Parameter values were taken from [4].

OX

$$v = k\mathrm{S} \quad \text{(S5)}$$

S: substrate of the reaction

Parameter	Value
k (s^{-1})	2.38E−05

The parameter value was adjusted to achieve the appropriate steady-state concentration of metabolites.

1-3. Comparison of minimal model and model with whole RBC metabolism

The minimal model with the above reactions and the whole RBC model both showed similar steady state values for metabolites.

Table 2. Comparison of steady state concentration of metabolites of original whole RBC model [1] and minimal model (t=10000 sec).

Name	Whole RBC model [1] (mM)	Simplified partial model (mM)
GSH	3.25	3.53
GSSG	0.46×10^{-2}	0.44×10^{-2}
GLCi	5.00 (fixed)	5.00 (fixed)
MgATP	1.49	0.98
MgADP	1.19	0.8
f23DPG	2.13	2.48
G6P	0.73	0.74
GDP	0.1	0.1
GL6P	5.24×10^{-6}	5.70×10^{-6}

1-4. Descriptions of diamide-mediated reactions

The following reactions represent the reactions that occur when the RBC is treated with diamide, thiol group oxidant. Simulation settings were set to 30% hematocrit, similar to the experimental study ([5]), by constructing a RBC compartment (where internal diamide is present) nested within a larger environment compartment (where external diamide, or ext_diamide is present). A was set to 0 at steady state, and was set to 1 to model the incubation of cells with diamide.

$$v = k \times [ext\_diamide] \times \frac{3}{7} \times A \quad \text{(S7)}$$

$$k = 1.27 \times 10^{-3} \text{ ([6])}$$

Once inside the cell, diamide rapidly reactions with GSH to form GSSG. (main text, Table 1 equation 2; [7]).

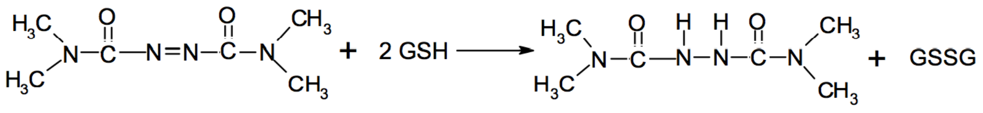

(S8)

When first simulating the model with the given reactions, we found that GSH was only slightly decreased, and it quickly returned to its initial levels (Figure 2, grey). Since the rate of direct diamide reduction with GSH was high, we assumed that there

was an alternative diamide-mediate pathway that created a long-lasting effect on GSH metabolism.

A survey on the effects of diamide revealed the following:

- A significant amount of GSH may be reversibly bound to protein, resulting in formation of mixed disulfides between GSH and protein sulfhydryl groups to form S-glutathionylated proteins (PSSG), under oxidative stress ([8])
- The formation of bonds is reversible after removal of oxidative stress ([9])
- In RBCs, diamide has been shown to induced mixed disulfide bonds between GSH and Hb, and also membrane skeletal proteins ([10], [11])

Given the above, we decided to incorporate these effects of diamide into the model so that there would be two pathways of GSH consumption. The reaction scheme is as shown below.

$$\mathrm{GSH+PSH} \xrightarrow{k3} \mathrm{PSSG} \quad \text{(S9)}$$

$$\mathrm{PSSG+GSH} \xrightarrow{k4} \mathrm{PSH+GSSG} \quad \text{(S10)}$$

As the parameters were not available in literature, we estimated these parameters by conducting a parameter analysis and comparing it with the time course observed from the previous experiment for the control RBC treated with diamide ([5]).

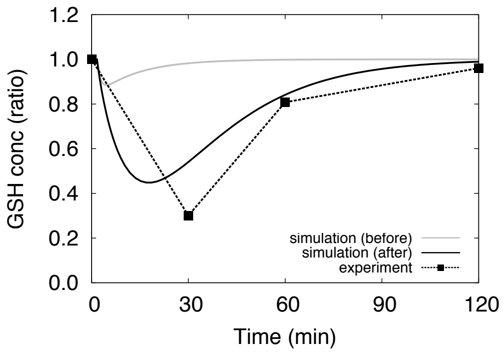

Figure 2. Comparison of GSH behavior in diamide-treated RBCs. The y-axis represents the GSH concentration relative to the initial concentration. Grey solid lines represent simulation results of the model without reactions S9 and S10. Black solid lines represent the results of the model that have incorporated these reactions, and the black dotted lines represents the experimental results from [5].

2. Detailed description of the band 3 clustering model incorporating spectrin interactions

The prototype model for band 3 clustering was extended to include the cytoskeletal components that characterize the RBC membrane.

2-1. Descriptions of modeling the cytoskeletal spectrin network compartment

In the model incorporating the spectrin cytoskeletal network, in addition to regular vacant voxel species, we assigned certain vacant voxel species to be specific to holding spectrin/bound band 3 species (main text Figure S2). Therefore, for each band 3 state (e.g. band 3, oxidized band 3, phosphorylated band 3, clustered band 3) there exists different species. For example for band 3 of natural state, band 3 occupying a vacant voxel (freeBand3), band 3 occupying a vertex voxel (BoundBand3), and band 3

occupying an edge voxel (Spectrin_band 3) due to its infrequent hopping between adjacent compartments (hop diffusion) over spectrin, can exist.

Normally the diffusion of diffusing freeBand3 is confined within the spectrin domains, however occasionally such band 3 are known to hop into other compartments (Figure 3, top right). We have represented the hop-diffusion scheme with a two-step reaction involving formation of a spectrin associate band 3 species.

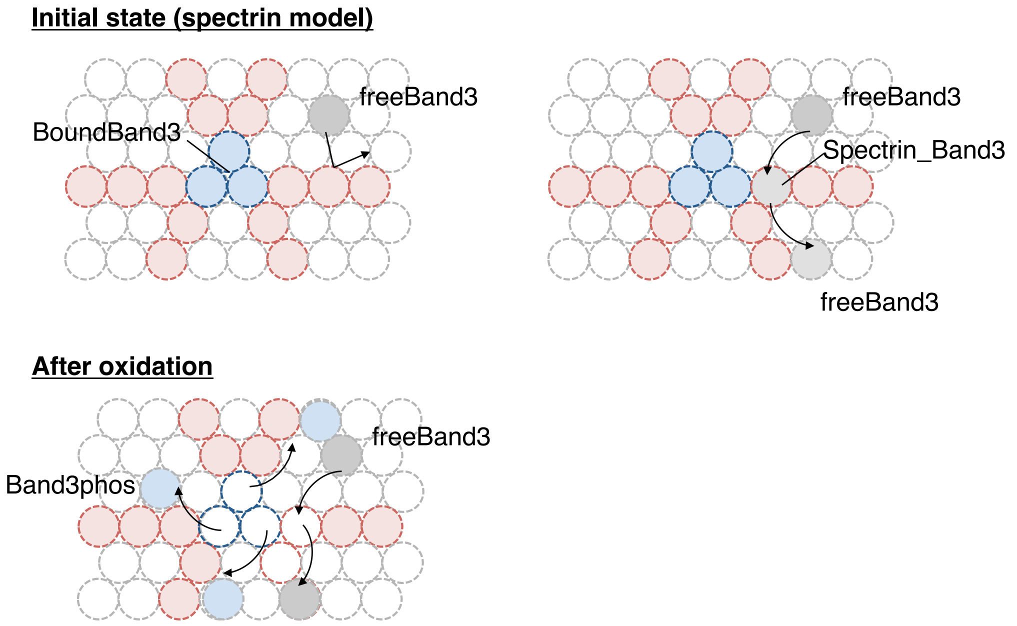

Figure 3. Schematic representation of how molecules behave in spectrin model. In the spectrin model, the initial spectrin meshwork mostly inhibits the free diffusion of spectrin non-bound band 3 (left). However, at a low probability, band 3 diffusing in confined regions enters a space between the cytoskeleton (here represented as the creation of a species, Spectrin_band3), and passes through the spectrin fence. When oxidation occurs, since sequential phosphorylation promotes the dissociation of BoundBand3 from spectrin, and thus results in a break in the structure, there is an increase in the number of band 3 molecules that can freely diffuse over domains (bottom).

The reaction scheme, equations, and parameters for the hop-diffusion reaction are given below.

$$\text{freeBand3} + \text{spectrin} \xrightarrow{p} \text{Spectrin\_Band3} \quad (\text{S11})$$

$$\text{Spectrin\_Band3} \xrightarrow{k} \text{Spectrin+Band3} \quad (\text{S12})$$

Parameter	Value	Reference
p	0.008	fitted to [12]
k	100 M s^{-1}	fitted to [12]

Once the RBC is treated with diamide, band 3 species (included BoundBand3) are oxidized, phosphorylated, and BoundBand3 become freely diffusing Band3phos molecules which diffuse away from their original positions. Since the tethering BoundBand3 is absent and replaced by a vacant compartment (vertex), the neighboring spectrin is also removed form its original position and replaced by a vacant compartment (edge). Thus oxidation induces a zipper like removal of the spectrin network. The reaction scheme, equations, and parameters for these reactions after oxidation are given below.

$$\text{spectrin} + \text{vertex} \xrightarrow{k1} \text{edge} \quad (\text{S13})$$

$$\text{edge} + \text{spectrin} \xrightarrow{k2} \text{edge} \quad (\text{S14})$$

Parameter	Value	Reference
k1	0.01	approximated
k2	0.0001	approximated

By incorporating these reactions representing the properties of the membrane at steady state, and the dissociation of the spectrin cytoskeleton upon oxidative treatment, we were able to more closely reproduce the effects of oxidation *in silico*, and assess the

how band 3 clustering may be regulated by the spatial organization of the RBC membrane.

References

1. Kinoshita A, Tsukada K, Soga T, Hishiki T, Ueno Y, et al. (2007) Roles of hemoglobin Allostery in hypoxia-induced metabolic alterations in erythrocytes: simulation and its verification by metabolome analysis. The Journal of biological chemistry 282: 10731–10741.
2. Mulquiney PJ, Kuchel PW (1999) Model of 2,3-bisphosphoglycerate metabolism in the human erythrocyte based on detailed enzyme kinetic equations: computer simulation and metabolic control analysis. The Biochemical journal 342 Pt 3: 597–604.
3. Buckwitz D, Jacobasch G, Kuckelkorn U, Plonka A, Gerth C (1990) Glucose-6-phosphate dehydrogenase from Plasmodium berghei: kinetic and electrophoretic characterization. Experimental parasitology 70: 264–275.
4. McIntyre LM, Thorburn DR, Bubb WA, Kuchel PW (1989) Comparison of computer simulations of the F-type and L-type non-oxidative hexose monophosphate shunts with 31P-NMR experimental data from human erythrocytes. European journal of biochemistry / FEBS 180: 399–420.
5. Pantaleo A, Ferru E, Carta F, Mannu F, Simula LF, et al. (2011) Irreversible AE1 tyrosine phosphorylation leads to membrane vesiculation in G6PD deficient red cells. PloS one 6: e15847.
6. Tribble DL, Jones DP (1990) Oxygen dependence of oxidative stress. Rate of NADPH supply for maintaining the GSH pool during hypoxia. Biochemical pharmacology 39: 729–736.
7. Kosower NS, Kosower EM, Wertheim B, Correa WS (1969) Diamide, a new reagent for the intracellular oxidation of glutathione to the disulfide. Biochemical and biophysical research communications 37: 593–596.
8. Giustarini D, Rossi R, Milzani A, Colombo R, Dalle-Donne I (2004) S-glutathionylation: from redox regulation of protein functions to human diseases. Journal of cellular and molecular medicine 8: 201–212.

9. Haest CWM, Kamp D, Deuticke B (1979) Formation of disulfide bonds between glutathione and membrane SH groups in human erythrocytes. Biochimica et biophysica acta 557: 363–371.
10. Dafré AL, Reischl E (1998) Oxidative stress causes intracellular reversible S-thiolation of chicken hemoglobin under diamide and xanthine oxidase treatment. Archives of biochemistry and biophysics 358: 291–296.
11. Rossi R, Giustarini D, Milzani A, Dalle-Donne I (2006) Membrane skeletal protein S-glutathionylation and hemolysis in human red blood cells. Blood cells, molecules & diseases 37: 180–187.
12. Tomishige M, Sako Y, Kusumi A (1998) Regulation mechanism of the lateral diffusion of band 3 in erythrocyte membranes by the membrane skeleton. J Cell Biol 142: 989–1000.
