# Supplementary figures and images for: Particle Simulation of Oxidation Induced Band 3 Clustering in Human Erythrocytes
Source: PLoS Comput Biol. 2015 Jun 5;11(6):e1004210. doi: 10.1371/journal.pcbi.1004210 (PMC4457884; doi:10.1371/journal.pcbi.1004210)

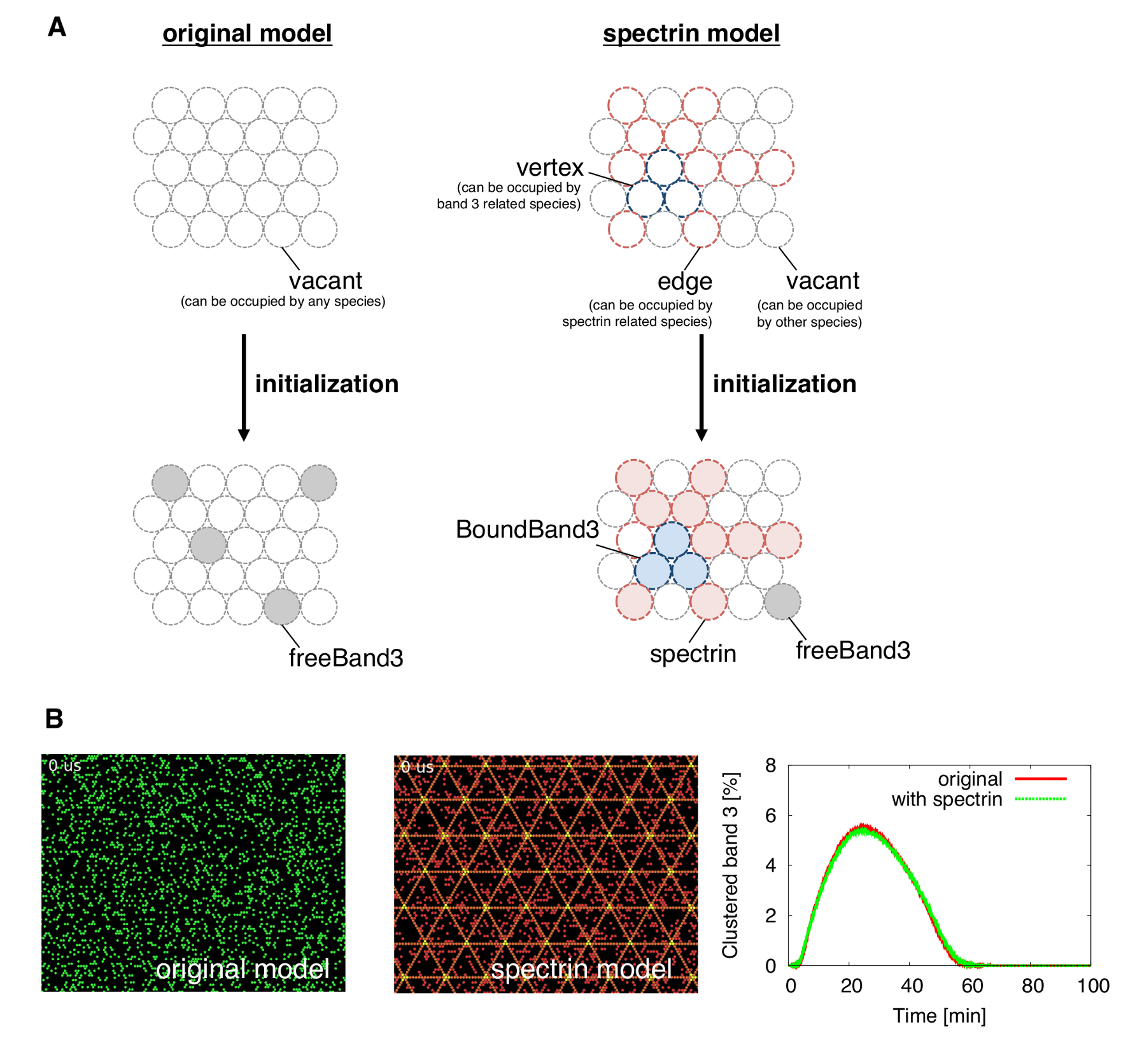

Supplement: S1 Fig — (A) Visualization of model structure in the original model with homogenously distributed Band 3, and model with spectrin, spectrin-bound Band 3 (BoundBand3), and corresponding vacant compartment species (edge and vertex). (B) Comparison of visual output at initial state (left), and levels of clustered band 3 (right) in the original band 3 model, and spectrin model with same reactions but using vertex and edge compartments. (TIF) [file pcbi.1004210.s002.tif]

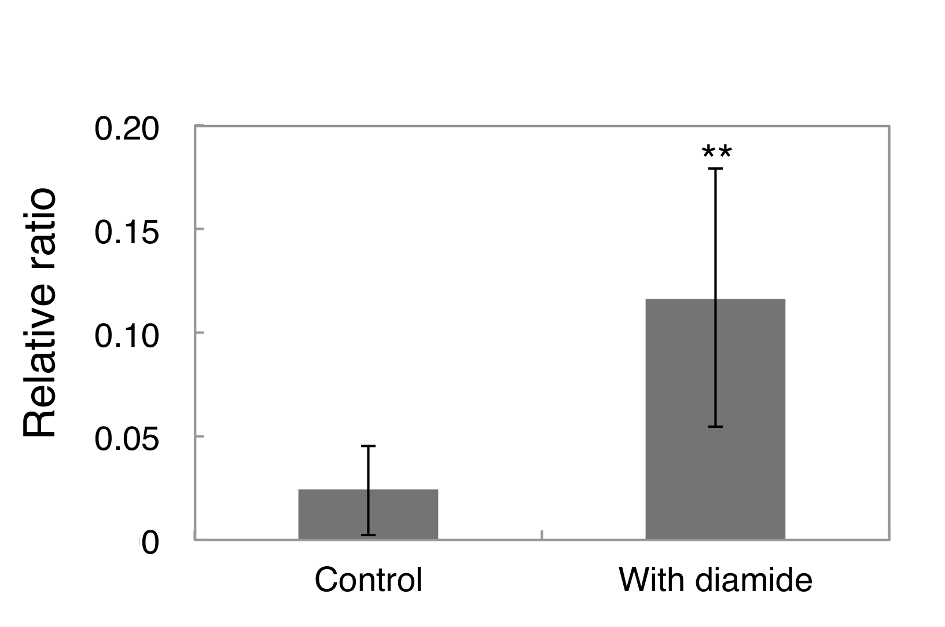

Supplement: S2 Fig — The x-axis represents the number of cells with inhomogeneous fluorescence distribution divided by the total number of cells. Cells were counted in 10–15 fields and data was obtained from >10 cells in each field. The analyses of data are shown as mean±SD of three independent experiments. Statistical analysis was performed between the control and the diamide-treated cells (n > 10, **p < 0.01 by t-test). (TIF) [file pcbi.1004210.s003.tif]

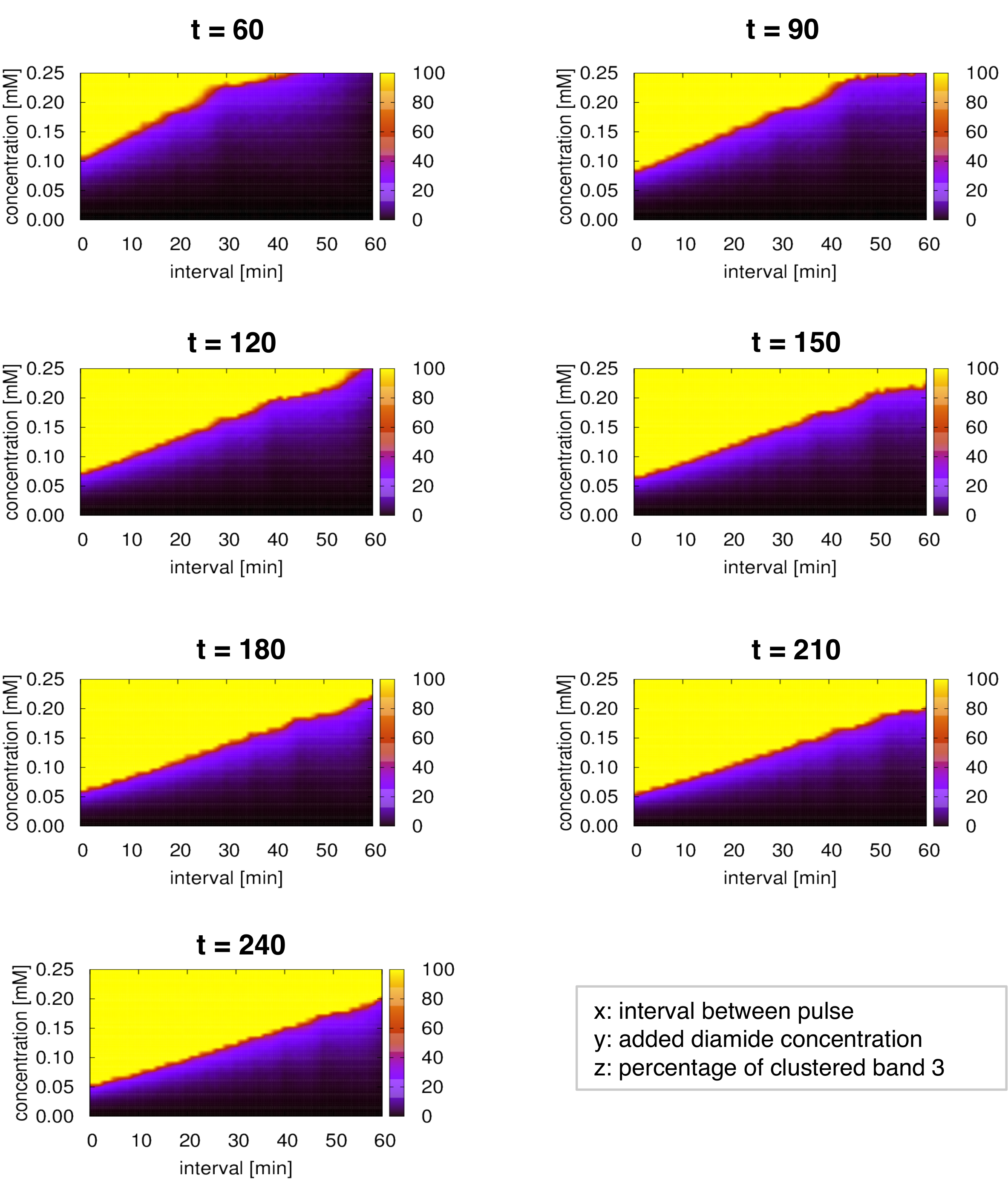

Supplement: S3 Fig — The x-axis represents the interval between pulses, y-axis represents the added diamide concentration, and z-axis represents the percentage of clustered band 3. (TIF) [file pcbi.1004210.s004.tif]

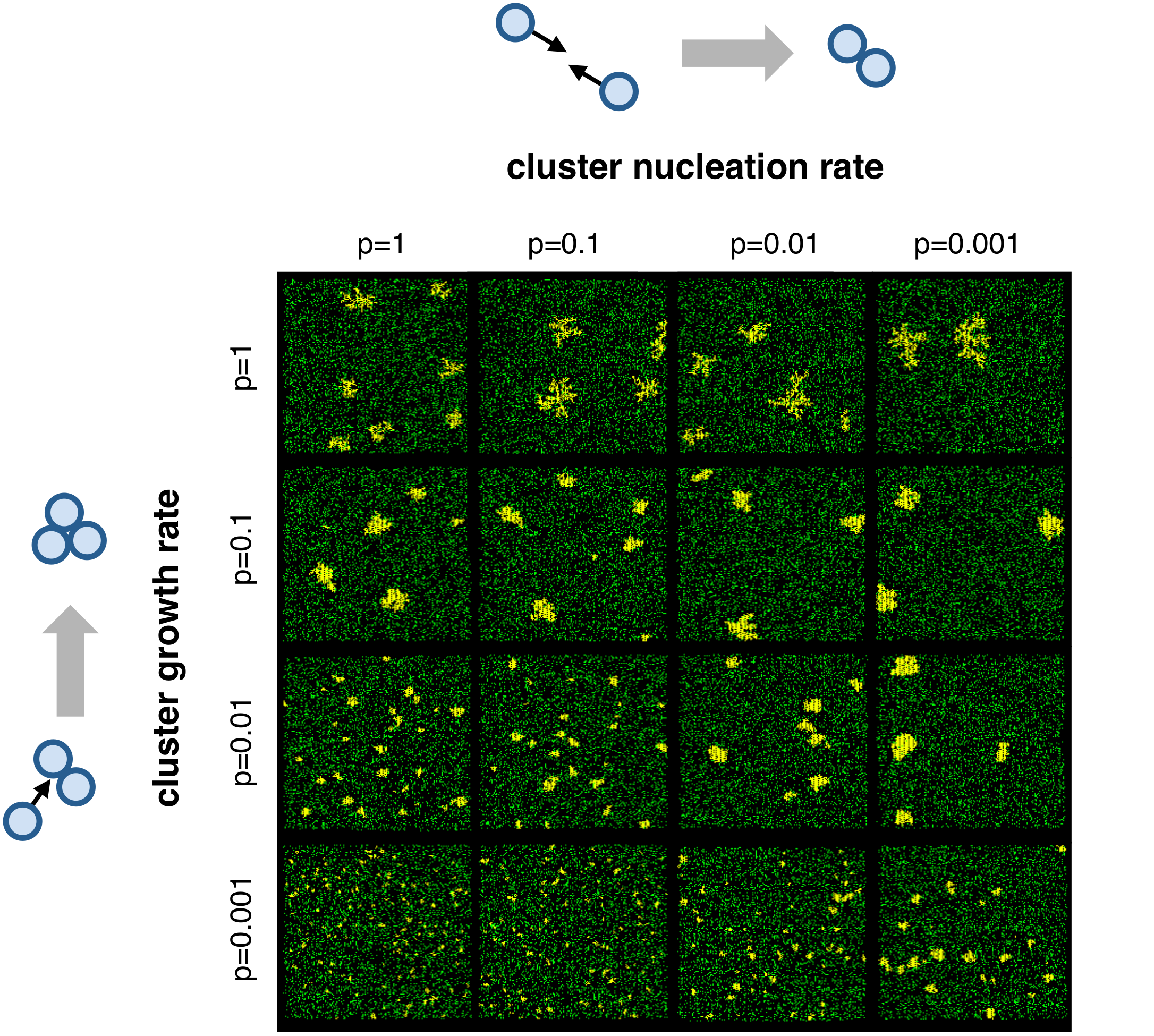

Supplement: S4 Fig — Images of cluster formation at t = 30 min. The x-axis represents the probability of molecules forming a new cluster upon collision, and the y-axis represents the probability of molecules adding on to a pre-existing cluster. (TIF) [file pcbi.1004210.s005.tif]
